# Absence of Claudin 11 in CNS Myelin Perturbs Behavior and Neurotransmitter Levels in Mice

**DOI:** 10.1038/s41598-018-22047-9

**Published:** 2018-02-28

**Authors:** Kathleen J. Maheras, Marcello Peppi, Farhad Ghoddoussi, Matthew P. Galloway, Shane A. Perrine, Alexander Gow

**Affiliations:** 1 Center for Molecular Medicine and Genetics, Wayne State University School of Medicine, Detroit, MI 48201 USA; 2 Department of Anesthesiology, Wayne State University School of Medicine, Detroit, MI 48201 USA; 3 Department of Psychiatry and Behavioral Neurosciences, Wayne State University School of Medicine, Detroit, MI 48201 USA; 4 Carman and Ann Adams Dept of Pediatrics, Wayne State University School of Medicine, Detroit, MI 48201 USA; 5 Dept of Neurology, Wayne State University School of Medicine, Detroit, MI 48201 USA

## Abstract

Neuronal origins of behavioral disorders have been examined for decades to construct frameworks for understanding psychiatric diseases and developing useful therapeutic strategies with clinical application. Despite abundant anecdotal evidence for white matter etiologies, including altered tractography in neuroimaging and diminished oligodendrocyte-specific gene expression in autopsy studies, mechanistic data demonstrating that dysfunctional myelin sheaths can cause behavioral deficits and perturb neurotransmitter biochemistry have not been forthcoming. At least in part, this impasse stems from difficulties in identifying model systems free of degenerative pathology to enable unambiguous assessment of neuron biology and behavior in a background of myelin dysfunction. Herein we examine myelin mutant mice lacking expression of the *Claudin11* gene in oligodendrocytes and characterize two behavioral endophenotypes: perturbed auditory processing and reduced anxiety/avoidance. Importantly, these behaviors are associated with increased transmission time along myelinated fibers as well as glutamate and GABA neurotransmitter imbalances in auditory brainstem and amygdala, in the absence of neurodegeneration. Thus, our findings broaden the etiology of neuropsychiatric disease to include dysfunctional myelin, and identify a preclinical model for the development of novel disease-modifying therapies.

## Introduction

The disconnection hypothesis developed by Carl Friston^1^ to account for the symptomatology of schizophrenia posits that aberrant behavior stems from disrupted communication between distributed neural circuits. An evolutionarily conserved feature of such networks is their organization into iterative feed forward-feedback loops, which process and integrate novel sensory information with existing engrams in increasingly extensive and parallel pathways. Signal processing eventually culminates in network refinement and yields specific, directed behavioral responses to stimuli^2^. Arguably, departures from normal behavior tend to emerge when sensory information is partially lost, discombobulated or temporally displaced.

To understand mechanisms underlying behavioral abnormalities in the context of the Friston hypothesis, most studies investigate neuron-specific etiologies, such as neurotransmitter imbalances, aberrant neurogenesis and other forms of disrupted neuron biology. On the one hand, associations between neurotransmitters and neurologic disease arose early from the recognition of structural similarities between serotonin and ergot alkaloids, which cause mental disturbances^3^. On the other hand, abnormal neuron biology arising during development or from injury, can alter signal processing/integration^4–8^. For example, precocious neuronal connectivity or supernumerary synapses are thought to underlie behavioral phenotypes in autism and epilepsy^9–13^.

Human and animal studies have documented neurochemical changes associated with behavioral phenotypes including anxiety, depression and neuropsychiatric illnesses^14–21^. Moreover, these studies have led to the current pharmacotherapeutic framework for developing disease-modifying therapies that restore neurotransmitter homeostasis^22–27^. Yet despite a plethora of marketed drugs, substantial numbers of medicated patients suffer breakthrough symptoms and additional serious side effects^28^. While there may be simple reasons for these negative patient outcomes, it is also possible that our present understanding of behavioral diseases should be broadened to consider additional etiologies.

In this regard, the inclusion of myelin dysfunction as an etiology of psychiatric disorders has been proposed for decades as a disconnection syndrome^29–32^ and the clinical literature is replete with studies of patients with white matter diseases, leukodystrophies and multiple sclerosis (MS), exhibiting neuropsychiatric symptoms^33–38^. While few investigators would disavow the importance of myelin for signal coherence and temporal connectivity in neural circuits, consideration of white matter dysfunction as an etiology for psychiatric diseases is routinely discounted in favor of the widely-accepted pharmacotherapeutic framework.

Perhaps the crux of a broader understanding of psychiatric etiologies is an appreciation of the intimate relationships between cells in the CNS; for example, damage to oligodendrocytes or other glial cells invariably leads to neuronal damage which causes abnormal behavior^39,40^. Ultimately, consideration of myelin dysfunction as a direct cause of behavioral changes is difficult without model systems in which myelin dysfunction arises in the absence of detectable neuronal injury or loss. To this end, we investigate loss-of-function behavioral phenotypes in *Claudin11* (*Cldn11*) knockout mice, which fail to assemble tight junctions in CNS myelin.

Previously, we demonstrated the molecular function of claudin 11 in CNS. The absence of this protein alters myelin passive properties in the absence of neurodegeneration^41–45^. Herein, we report that *Cldn11*-null (*Cldn11*^*−/−*^) mice exhibit central auditory deficits, reduced anxiety-like behavior and neurotransmitter imbalances; glutamate in the brainstem and GABA in the amygdala/ventral hippocampus. Together, these data not only facilitate assessment of myelin dysfunction as an etiology for behavioral disease, but also highlight complex abnormalities arising from a monogenic defect. In this regard, *Cldn11*^*−/−*^ mice may exemplify a multimodal phenotype in distributed brain regions that is refractory to simple monotherapies aimed at restoring GABA or glutamate neurotransmitter homeostasis.

## Materials and Methods

The datasets and methods used during and/or analyzed during the current study are available from the corresponding author on reasonable request. The numbers of mice used for quantitative measurements are included in the figure legends. Qualitative data shown in the figures are representative of the data we use for quantitation.

### Approvals and study design

The Department of Laboratory and Animal Resources at Wayne State University oversaw the husbandry of mice in this study. All experiments were performed in accordance with an Institutional Animal Care and Use Committee protocol approved by the Wayne State University Animal Investigation Committee (A 04-02-09; A 03-01-12; A 01-11-15). Mice were maintained on a 12 h light/dark cycle (on at 06:00) with standard rodent chow and water available *ad libitum*. Transgenic mice [*Tg*^+^; Tg(Cldn11)605Gow; MGI:5306245] as previously described^46^ were crossed with *Cldn11*^*−/−*^ mice.

### Transgene generation and mouse husbandry

Generation of a 20 kilobase mouse *Cldn11* genomic transgene [*Tg*^+^; Tg(Cldn11)605Gow; MGI:5306245], comprising 5 kb upstream of the transcription start site to 1.9 kb downstream of the first polyadenylation site in exon 3, was previously described^46^. The genomic fragment was cloned between two *Not I* sites in a *pUC18* – based vector, excised and phenol:chloroform-purified from an agarose gel for male pronuclear injection^47^. Initial characterization of the transgene was performed using 6 independent lines (#5, 8, 9–12). Double mutant *Cldn11*^+/−^*::*Tg(Cldn11)605Gow^*Tg/*+^ (*Cldn11*^+/−^*::Tg*^+/−^) mice were generated for each line by breeding to *Cldn11*^*−/−*^ females and thereafter sib-mated to generate *Cldn11*^*−/−*^*::Tg*^+/−^ and *Cldn11*^+/−^*::Tg*^+/−^ mice for characterization. Line #12 was selected for characterization in the current study. Mice were genotyped from toe/tail biopsies digested overnight in 0.5 mg/ml proteinase K (Sigma, St Louis, MO) in DirectPCR (Viagen, Los Angeles, CA) per the manufacturer recommendations. To genotype the endogenous *Cldn11* gene, we used a standard PCR protocol with primers 5′-GTCGCAGCAGTGCTCGCAGCCGCTC-3′, 5′-GTCCTTACCTGGAAGGATGAGGATG-3′ and 5′-ATGTGCTGCAAGGCGATTAAGTTGG-3′. To genotype the Tg(Cldn11)605Gow allele, quantitative PCR (StepOne cycler, Thermo Fisher) was used to determine copy number using the following primers: 5′-ACATGGTGGGAAATCATATACACTATTGTTAA-3′ and 5′-ACTGGAGTGTCTCTTTGGTCGATTA-3′ and TaqMan probes 5′-CAGGTACGTACCCAAGC-3′ and 5′-TCCCAGGTACCCAAGC-3′ (Thermo Fisher).

### Behavioral tests

Mice were habituated to the experimental room 1 h prior to all behavioral tests. Behavioral tests were conducted between 0600–0900. For open field testing, a 61 × 61 × 31 cm black matted Plexiglas arena (Formtech Plastics, Oak Park, MI) was used. On two consecutive days, mice were placed in the center of the arena and video recorded for 5 min. Videos were analyzed post-hoc using Ethovision software (version 8.5, Noldus, Leesburg, VA) and all data values averaged across both days of testing. Center square percentages were derived from average mouse body length distances from the edges of the arena: 0.5 body lengths, the center square is 83% of total arena area; 1, 66%; 1.5, 48%; and 2, 32%. Marble burying tests were carried out as previously described^48^ using 15 marbles in a 3 × 5 grid pattern. At the end of 30 min, marbles buried 2/3 of the way with bedding were tallied. Tail suspension was carried out for 6 min following previously published methods^49^ excluding white noise. Videos were manually scored by a blinded reviewer for the amount of time spent immobile.

### Antibodies

The antibodies used in this study were: Rb anti-NF-L (Cell Signaling, Danvers, MA); Ms anti-MBP (Sternberger, Covance, Princeton, NJ); Rb anti-Calbindin D28K (Sigma); Ms anti-claudin 11 (clone 37E3)^41^; Rb anti-connexin 26 (Zymed, Thermo Fisher, Waltham, MA); Gt anti-MsIgG1 Alexa488 (Molecular Probes, Eugene, OR); Gt anti-MsIgG2b Alexa488 (Molecular Probes); Gt anti-RbIgG Alexa568 (Molecular Probes); Gt anti-MsIgG1 Alexa568 (Molecular Probes). Nuclei were labeled with DAPI (4′6′-diamidino-2-phenylindole, Sigma).

### Cryostat sectioning

Adult *Cldn11*^*−/−*^*::Tg*^+/−^ mice were injected i.p. with 400 mg/kg avertin, and monitored for the absence of pain reflexes, signifying deep anesthesia. The chest cavity was exposed and mice were perfused transcardially with 4% PFA in 0.1 M phosphate buffer pH for 15 min. Brains were dissected and placed in 25% sucrose in PBS overnight at 4 °C. The sucrose solution was exchanged the following morning, and after 6 h the brains were removed from the sucrose solution, blotted dry, placed in a plastic cryomold (Tissue-tek Andwin Scientific, Schaumburg, IL), covered with OCT (Andwin Scientific) and frozen in a 2-methyl butane (Thermo Fisher, Waltham, MA)/dry ice slurry and stored at −80 °C.

**Figure 1:**
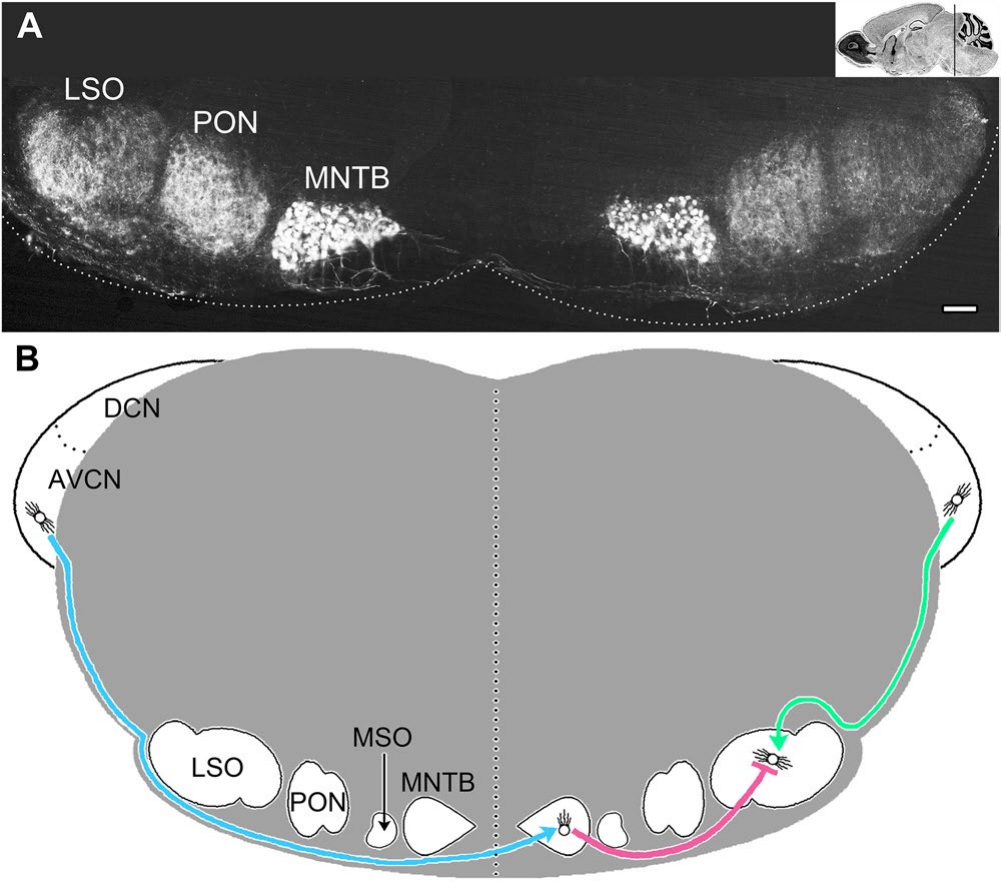
Key nuclei and fiber tracts for binaural transmission in adult mouse auditory brainstem. **(A)** Calbindin staining labels most of the key bilateral brainstem nuclei in the superior olivary complex (SOC). Principal neurons of the medial nuclei of the trapezoid body (MNTB) are nearest to the midline and form the calyxes of Held. The lateral superior olive (LSO) are the lateral nuclei, and are major signal integrators of binaural signals from the cochleae. The medial superior olive (MSO, not labeled) is poorly developed in mice (approximately 200 neurons) and may only play a minor role in integrating binaural signals. The periolivary nuclei (PON) are likely modulatory but their dispersed cell bodies suggests they generate signals with insufficient synchrony to be detected in far field recordings such as ABRs^88^. **(B)** Lower brainstem schematic of three major myelinated fiber tracts and their connections that transmit binaural information to principal cell integrators in the LSO. Only the fiber tracts from the left cochlea to the right LSO (comprising the contralateral pathway), and the right cochlea to the right LSO (the ipsilateral pathway), are shown for clarity. The AVCN – MNTB tract of the trapezoid body (blue) is comprised of large diameter axons and transmits contralateral signals using the excitatory neurotransmitter glutamate; the MNTB – LSO tract (pink) is comprised of intermediate size axons and relays ipsilateral inhibitory signals (glycinergic) in adults; the AVCN – LSO tract (green) is comprised of small axons and transmits excitatory ipsilateral signals (glutamatergic). Each of these nuclei are signal generators for ABR waves. Thus, the cochlear nucleus is associated with wave II, the MNTB with wave III and the SOC with wave IV. Wave V arises from the transmission of signals to the inferior colliculus. Scale bar, 200 μm. Insert in A: Image Credit, Allen Institute.

Tissue blocks were cut into 10 µm sections using a cryostat at −20 °C (Shandon Electric Cyrotome, Thermo Fisher) and every fifth section (50 µm) was thaw-mounted on Superfrost slides (Thermo Fisher) and stored at −80 °C for subsequent immunolabeling. To quantify the extent of myelination for trapezoid body fibers, sagittal sections were cut between the midline and the medial nucleus of the trapezoid body (MNTB) principal neuron cell bodies in the calyx of Held (Fig. 1; decussating, presynaptic afferents from the contralateral cochlear nucleus, 1 section/mouse), as well as within the calyx of Held (synapsing principal cells, 3 sections/mouse spaced 50 µm apart). Sectioning and immunofluorescence staining of the lateral wall of the cochlear duct is previously described^42^.

### Immunofluorescence labeling

Slides were thawed from −80 °C in PBS for 5 min and permeabilized with methanol (Thermo Fisher) for 20 min at 4 °C. After washing in PBS, slides were blocked in 2% goat serum/TBSGBA for 1 h. Primary antibodies NF-L (Cell Signaling) and MBP (Sternberger, Covance) in block solution were incubated over night at room temperature with rocking. Slides were washed with PBS, incubated with secondary antibodies conjugated to Alexa586 and Alexa488 (Molecular Probes) and DAPI (Sigma) for 3 h at room temperature, and mounted in Vectashield (Vector Laboratories, Burlingame, CA). Transversely-sectioned axons were considered myelinated when neurofilament staining was circumscribed by MBP (≥270°). Images were assembled using Photoshop CS6 (Adobe Systems, San Jose, CA).

### Auditory fiber tract tracing

Tract tracing was performed using published methods with minor variations^50^. Briefly, mice were perfused with 10 ml of freshly oxygenated artificial cerebral spinal fluid (aCSF: 126 mM NaCl, 3 mM KCl, 2 mM CaCl_2_, 2 mM MgSO_4_, 1.25 mM NaH_2_PO_4_, 26 mM NaHCO_3_, 10 mM dextrose, pH 7.4), the brains dissected and cut into 2 mm coronal sections (see Supplementary Methods).

Auditory brainstem slices were bathed in carbox-aCSF for microinjection under a stereomicroscope (Leica, Solms, Germany). Needle tips were inserted 0.2–0.8 mm below the surface of the tissue and 30 nl of Alexa488-Dextran (Molecular Probes) was injected into target nuclei or fiber tracts. The dye was injected unilaterally in 1–3 bolus deposits for labeling of connections between the cochlear nucleus and either MNTB or LSO. After 4–6 h incubation in carbox-aCSF at room temperature, the tissue slices were fixed overnight with 4% paraformaldehyde (Sigma). Vibratome sections (70 µm) were cut (Vibratome Series 1000 Plus) and labeled with anti-Calbindin D28K antibodies (Sigma) at 4 °C and mounted in Vectashield (Vector Laboratories). Dextran-labeled fibers from the cochlea nucleus that entered the contralateral MNTB were analyzed, as were ipsilateral dextran-labeled fibers entering the LSO.

### Confocal imaging

Confocal fluorescence image stacks of dextran-labeled axons were captured using a Leica DM5500 microscope equipped with Plan Apo 20× and 40× dry lenses, Yokogawa spinning disk confocal using an Argon/He/Ne laser and a Hamamatsu Orca ER^2^ digital camera. Confocal image stacks were acquired using a 63× Plan Apo oil-immersion objective lens (*n*.*a*. = 1.4) in 0.2 µm increments through 70 µm vibratome sections. After collection, stacks were quantified using ImageJ software. The location of each fiber within a stack was determined and analyzed only if its entire length could be tracked with confidence. Mini stacks of these fibers were collapsed to single through-focus images. For ipsilateral fibers, 20–30 measurements of diameter were recorded at equidistant intervals for axons entering but not exiting the lateral superior olivary nucleus (LSO). To distinguish contralateral auditory fibers from pyramidal fibers, only axons attached to a calyx of Held were analyzed, and 20–30 equidistant measurements were recorded beginning 10 µm from the calyx. Average axon diameters (*n* ≥ 3 mice) used for histograms with binning = 0.2 µm and a Gaussian curve fit.

### Auditory pathway neurophysiology

Monaural and binaural ABRs were acquired using two channel recordings with reference (inverting) subdermal mastoid electrodes (Grass, Natus Neurology, Middleton, WI) and a non-inverting vertex electrode^42^. Binaural interaction components were derived from binaural ABRs^51^. Mice were habituated to the experimental room 1 hr prior to testing; all auditory behavioral paradigms were conducted between 1300–1700. ABRs were acquired as previously described^42^ using 100,000× pre-amplification from 102 µs pure tone pips at 8, 16, or 32 kHz (Sepwin software version 5.1, Intelligent Hearing Systems, Miami, FL). Hearing thresholds were determined by identifying the lowest stimulus intensity in which wave I was identified.

Individual wave latencies were measured at the immediate right side of the crest for each waveform. Interpeak latencies were obtained by subtracting raw wave latencies to obtain total (waves V – I), central (waves V – II), and peripheral (waves II – I) interpeak latencies. Amplitude was measured by convention from wave maxima to the immediately following trough minima. Area under the curve was calculated by connecting the baselines drawn between the preceding and succeeding troughs.

Interaural level delays were recorded at 32 kHz (10–3,000 Hz band pass filter) using ABR stimulus parameters once a symmetrical hearing threshold of ≤40 dB SPL was confirmed. First, midline level delays were obtained by playing 70 dB SPL to the left ear alone, right ear alone, then simultaneously. Subsequently, left ear stimulus intensity was decreased to 68, 65, 60, 50, and 40 dB SPL in conjunction with or without a 70 dB SPL stimulus presented to the right ear. This process was reversed for decreasing stimuli to the right ear. Binaural ABR traces were post filtered 120–3,000 Hz to resolve waves IV and V for analysis.

All BIC traces were calculated by subtracting post-filtered left and right monaural ABR traces (μV weighted) from binaural ABR traces acquired from dichotic stimuli. For example, left ear BICs were derived by subtracting from the dichotic trace, the monaural right ipsilateral trace at 70 dB SPL and the monaural left ipsilateral trace at ≤70 dB SPL. This process was repeated for the right ear. All traces were post-filtered with predetermined high/low pass filters 10–500 Hz to optimize the interaction trough and reduce noise. Characteristics of the DN1 trough are used to determine the processed output from the superior olivary complex (SOC), which we verified to occur after the monaural signals arrived at the SOC (wave IV), but prior to signal generation at downstream nuclei (wave V).

### High field magic angle proton magnetic resonance spectroscopy (^1^H-MRS)

Mice were decapitated and whole brains immediately removed and placed on an ice-cold coronal matrix (ASI, Warren, MI) to cut 2 mm thick coronal slices. The slices were frozen on a dry ice slab and specific nuclei were harvested bilaterally using 1.5 mm diameter disposable biopsy punches (Miltex, San Mateo, CA) and stored at −80 °C for neurochemistry. Punches were placed in a Bruker zirconium rotor (2.9 mm diameter, 10 µL capacity) inside a Bruker magic angle spinning probe maintained at 4 °C and rotated at 4.2 ± 0.002 kHz at 54.7° relative to the static B_0_ field as previously described^52^. The samples were analyzed from 128 scan averages in a vertical wide-bore (8.9 cm) Bruker 11.7 T magnet with an AVANCE^TM^ DRX-500 spectrometer (Bruker Biospin Corp., Billerica, MA) as previously described^53^. The neurochemicals quantified were: acetate, alanine, aspartate, betaine, creatine, choline, gamma-aminobutyric acid (GABA), glutamine (Gln), glutamate (Glu), glycerophosphocholine (GPC), glutathione, myo-inositol, lactate, N-acetylaspartate (NAA), N-acetylaspartate-glutmate (NAAG), phenylethylamine (PEA), phosphocholine, succinate and taurine.

### Sampling details and statistics

For all experiments, mice were allocated to experimental groups by genotype based on rolling enrollment. For each experiment, mice were retrieved from WSU housing facilities and genotypes were masked by the investigator for experiments. Mice were manually selected at random. Sample sizes were predetermined, either from legacy pilot experiments or using sample sizes from our published studies or typical sample sizes from the literature.

Statistical analyses were performed using GraphPad Prism (version 5, La Jolla, CA) with the appropriate tests and significance values included in the figure legends. All statistical tests used were two-tailed and data are expressed as mean ± SEM with the number of independent observations, *n*, or the degrees of freedom indicated in the figure legends. QQ-plots demonstrating unimodal distributions of axon diameters in the SOC were generated using the ‘car’ package in RStudio (version 1.1.414)^54^. Dashed lines indicate the 95% confidence envelope obtained by parametric bootstrapping.

### Data availability

Reagents, mouse mutants and datasets generated and/or analyzed during the current study are available from the corresponding author on reasonable request.

## Results

In previous studies, we and others have characterized functions of claudin 11 tight junctions in three cell types: Sertoli cells in testis, basal cells in the Stria vascularis of the cochlea and myelin sheaths in the CNS^41–43,45,55–57^. Global knockout of the *Cldn11* gene has significant consequences in adult mice; Sertoli cells fail to support sperm maturation and basal cells fail to generate an endocochlear potential. In the CNS, multilamellar myelin sheaths lack the mesaxon, paranode and outer lamella tight junctions that circumscribe the intramyelinic compartment^41,45^, thereby increasing the vulnerability of compact myelin to electrochemical gradients and lowering membrane resistance. This vulnerability increases transmission time in a manner inversely proportional to axon caliber^43,44^.

A likely consequence of changes to the passive properties of compact myelin would be temporal dispersion of encoded signals. For signals converging on integration nuclei from multiple white matter tracts, temporal dispersion could distort information processing when the afferent axons are of different calibers, potentially giving rise to behavioral phenotypes. Importantly, we do not detect changes in myelin thickness, *g*-ratio, structural stability, or evidence of axon loss^41,43,45^; thus, we can interpret abnormal behavior in *Cldn11*^*−/−*^ mice in terms of temporal dispersion/signal loss and incomplete network refinement rather than degenerative pathology in neurons^58^.

In this light, we postulated that behavioral deficits might arise when signal transmission to integration/processing nuclei depend on large and small caliber myelinated afferents. We tested this hypothesis in the spatially-restricted circuit of the superior olivary complex (SOC), which plays a major role in integrating auditory signals from the left and right ears in mammals^59,60^. The SOC is a group of ventral bilateral nuclei in the brainstem with limited neural plasticity in adulthood, including the medial nucleus of the trapezoid body (MNTB), the medial superior olivary nucleus (MSO, a minor nucleus in mice) and the lateral superior olivary nucleus (LSO).

### The Tg(Cldn11)605Gow transgene restores peripheral hearing

A major barrier to studying the auditory pathway in global *Cldn11*^*−/−*^ mice is the profound deafness emerging by 2 months of age^42^. Accordingly, we generated a genomic *Cldn11* transgene (Supplementary Fig. S1, *Tg*^+/−^) to rescue loss-of-function phenotypes in *Cldn11*^*−/−*^ mice. This transgene is expressed in Sertoli cells and cochlea basal cells. Confocal micrographs from the cochlea lateral wall of *Cldn11*^*−/−*^*::Tg*^+/−^ mice show transgene expression in basal cells that is indistinguishable from endogenous claudin 11 expression. Expression patterns for most of the other transgenic lines are similar. Transgene expression in wild type mice does not affect hearing thresholds or ABR wave latencies, demonstrating that several-fold supernormal expression of claudin 11^46^ is not detrimental to basal cells or cochlear function.

Crossing the Tg(Cldn11)605Gow transgene into the *Cldn11*^*−/−*^ background rescues hearing thresholds, as determined by pure tone ABRs at 8, 16 and 32 kHz in 4/6 transgene lines (lines #5, 8, 11 and 12; Supplementary Fig. S2), and confers normal outer hair cell function (DPOAEs). Transgenic lines #9 and #10 do not rescue hearing thresholds in *Cldn11*^*−/−*^*::Tg*^+/−^ mice, which remain similar to littermate *Cldn11*^*−/−*^ mice. We demonstrated low transgene expression in these two lines^46^, which likely also account for unabated deafness in the current study.

Despite rescuing peripheral deafness in *Cldn11*^*−/−*^ mice by 4/6 transgenic lines, the sensorineural phenotype of increased ABR wave V latency^42^ is undiminished and suggests that Tg(Cldn11)605Gow is not expressed in the CNS (Supplementary Fig. S3). Thus, the link between the two auditory deficits can be uncoupled, with the latter being caused by loss-of-function in CNS myelin^41,43,44^. To characterize this uncoupling and explore potential CNS phenotypes, we characterized *Cldn11*^*−/−*^ mice bred with Tg(Cldn11)605Gow line #12 transgenic mice (*Cldn11*^*−/−*^*::Tg*^+/−^) for comparison with littermate controls (*Cldn11*^+/−^*::Tg*^+/−^).

### The Tg(Cldn11)605Gow transgene is not expressed in the CNS

Similar to many genes, *Cldn11* harbors multiple enhancers in different locations of the gene that regulate cell type-specific expression. For example, putative Klf1/Gata and Pou3f4 enhancers approximately 1 kb and 4.8 kb (respectively) upstream of exon 1 appear to regulate expression in Sertoli cells and cochlear basal cells (A.G., unpublished)^46,61^. Both enhancers are within the Tg(Cldn11)605Gow transgene comprising 5.5 kb of upstream sequence.

Using transient transgenic mice, we have identified a major enhancer in proximity to exon 3 that drives robust expression of *Cldn11* in oligodendrocytes (Supplementary Fig. S4). Further, we demonstrate that the Tg(Cldn11)605Gow transgene is not expressed in brain at the levels of RNA or protein, which suggests the oligodendrocyte enhancer in the endogenous *Cldn11* gene is downstream of exon 3 [beyond the 3′ extent of Tg(Cldn11)605Gow]. Indeed, the Peterson lab^62^ have characterized a 631 bp enhancer located 2 kb downstream of exon 3 that is homologous to a conserved transcriptional regulatory network for oligodendrocyte myelin gene expression. Together, our data show the Tg(Cldn11)605Gow transgene rescues deafness and male infertility in *Cldn11*^*−/−*^ mice, but is not expressed by oligodendrocytes. Thus, it is likely that transgene expression neither alters CNS myelin properties characterized in *Cldn11*^*−/−*^ mice nor rescues brain-associated phenotypes.

### Small and large myelinated axons in mouse auditory brainstem

The auditory pathway below the inferior colliculus is a precisely-timed neural circuit that is convenient for neuroanatomical and electrophysiological analyses and has been characterized in extraordinary detail. Most of the prominent brainstem nuclei from the cochlear nucleus to the SOC can be visualized in a single coronal brain slice with antibodies against the calbindin D28K protein (Fig. 1A). Major fiber tracts connecting these nuclei are shown schematically in Fig. 1B.

**Figure 2:**
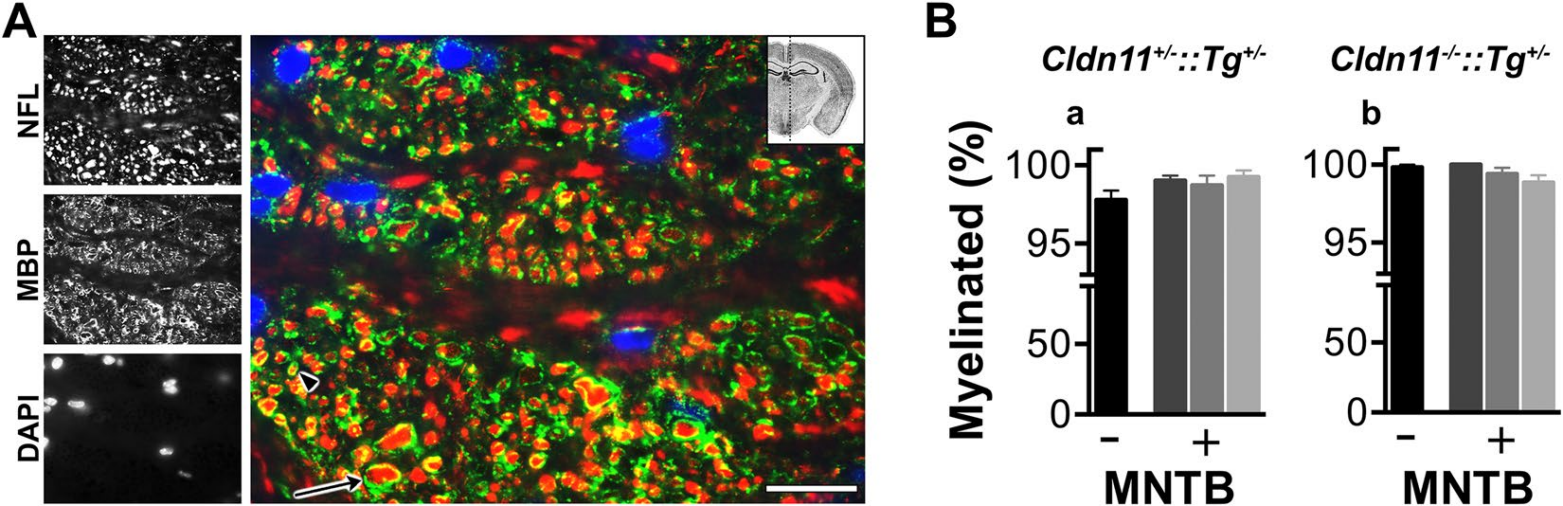
Myelinated axons in the auditory pathway. **(A)** Representative sagittal brainstem section of the trapezoid body fiber tract (see insert). Anti-neurofilament light chain (NFL, red) antibodies label all axons. The major structural myelin protein, myelin basic protein (MBP) labels myelin sheaths (green) and nuclei are labeled with DAPI (blue). Distinct transverse fiber bundles are shown, cut in cross-section. The arrowhead and arrow show small (1 μm) and large (5 μm) diameter transverse fibers. **(B)** Proportion of myelinated fibers traversing the trapezoid body tract in (Ba) *Cldn11*^+/−^*::Tg*^+/−^ and (Bb) *Cldn11*^*−/−*^*::Tg*^+/−^ mice. We quantified fibers from a single sagittal section/mouse (−), which was medial to MNTB principal neuron cell bodies in the calyx of Held and included contralateral afferents from the cochlear nucleus. We also quantified fibers from 3 sections/mouse, spaced 50 μm apart (+), within the MNTB and included calyx of Held principal neuron cell bodies. Data plotted as mean ± SEM; *n* = 3 fields/slide totaling ~150 axons. Scale bars, 20 μm. Insert in A: Image Credit, Allen Institute.

To ensure reliable signal dissemination (temporal fidelity) and rapid propagation speed, auditory brainstem fibers in most mammals are myelinated^63,64^. We confirmed this general feature in trapezoid body fibers (Fig. 1B), which connect the cochlear nucleus to the MNTB. Sagittal sections of this tract were labeled with antibodies against neurofilament to show axons and myelin basic protein for myelin sheaths (Fig. 2A). Small and large diameter axons are interspersed within the fiber bundles and we conservatively estimate 98.7% of the axons are myelinated in both control (Fig. 2Ba) and *Cldn11*^*−/−*^*::Tg*^+/−^ (Fig. 2Bb) mice.

**Figure 3:**
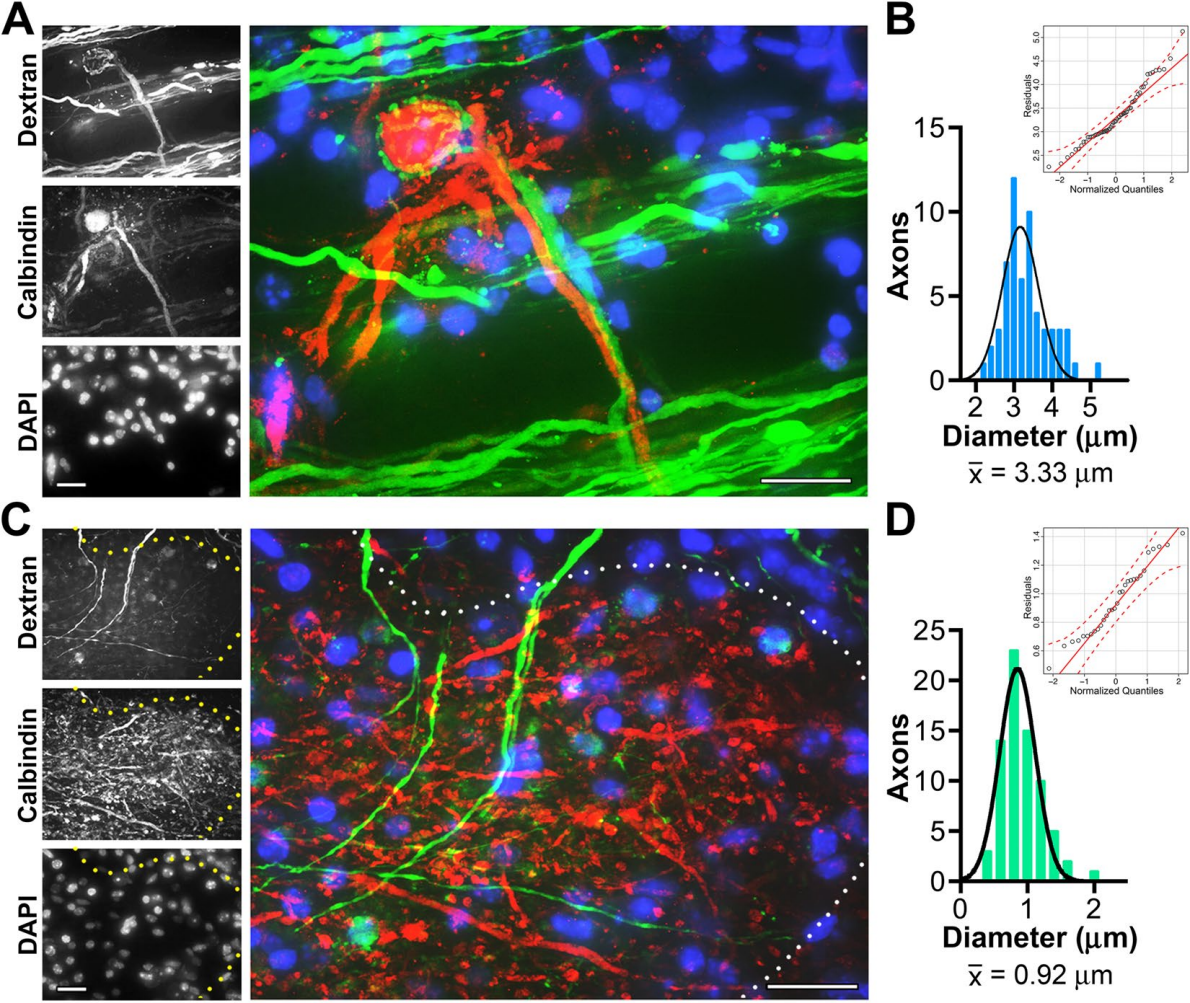
Morphometric analyses of auditory brainstem fiber tracts reveal contralateral axons are large diameter and ipsilateral axons are small. (**A**) Within the MNTB, a representative contralateral dextran labeled fiber (green) is seen forming the characteristic calyx of Held synapse around a Calbindin + MNTB principal cell (red). Nuclei are stained using DAPI (blue). (**B**) Distribution of contralateral diameters from dextran-dye labeled axons. The histogram is comprised of 59 axons from 3 mice, 0.2 μm bins, and fit with a normal Gaussian distribution. Mean contralateral fiber diameter is considered large: 3.3 μm. The Q-Q plot (inset) shows most residuals are distributed along the Gaussian diagonal within the 95% confidence envelope (parametric bootstrapping, red dashed lines) as expected for a unimodal distribution. **(C)** Representative ipsilateral fibers (green) are seen entering the LSO outlined by Calbindin staining (red, white dots). Nuclei are stained using DAPI (blue). **(D)** Distribution of ipsilateral diameters from dextran-dye labeled axons viewed entering the LSO. Histogram is comprised of 73 axons from 5 mice, 0.2 μm bins, and fit with a normal Gaussian distribution. Mean ipsilateral axon diameter is considered small: 0.91 μm and the Q-Q plot indicates a unimodal distribution. Scale bars, 20 μm.

The diameters of myelinated fibers in the auditory brainstem have been measured in birds and several mammalian species^64–68^. We used fluorodextran tract labeling of fibers between the cochlear nuclei and the SOC (Fig. 3A) to locate globular bushy cell axons from the anterioventral cochlear nucleus (AVCN) forming contralateral connections with MNTB principal cells at the calyx of Held synapse^69,70^. Axon diameter measurements of 59 contralateral axons from wild type mice approximate a normal distribution (Fig. 3B), with an average diameter of 3.3 ± 0.07 μm (*n* = 3), and coincide with recently published values^66^.

Conversely, spherical bushy cell axons from the AVCN form ipsilateral connections with the principal cells in the LSO (Fig. 3C). Measurements of 73 axons from wild type mice are also normally distributed (Fig. 3D) with an average diameter of 0.9 ± 0.03 μm (*n* = 5). Together, these data indicate that ipsilateral fibers are 3.7-fold smaller in diameter than contralateral fibers and transmission time will be disproportionately affected by the absence of claudin 11^44^. Indeed, we estimate transmission time along contralateral fibers of *Cldn11*^*−/−*^*::Tg*^+/−^ mice should increase approximately 20% compared to controls; however, the increases along ipsilateral fibers would likely exceed 40%.

### Abnormal monaural ABRs

**Figure 4:**
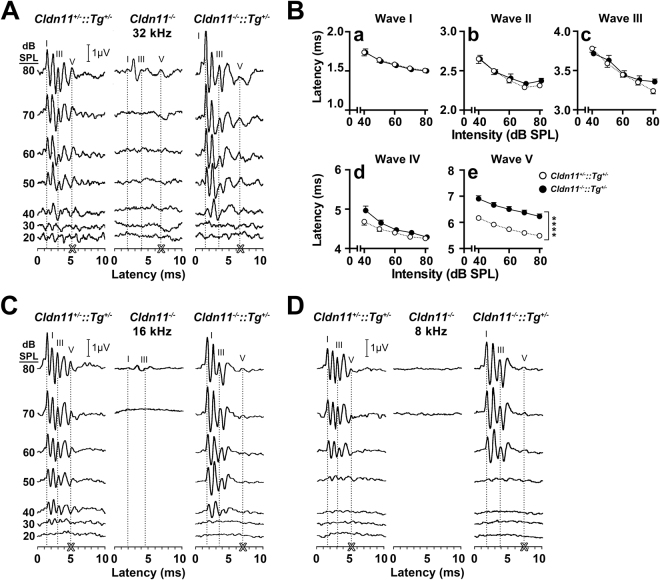
The Tg(Cldn11)605Gow transgene (*Tg*^+/−^) restores hearing thresholds, but not wave V latencies in *Cldn11*^*−/−*^*::Tg*^+/−^ mice. **(A)** Representative ABR series from *Cldn11*^+/−^*::Tg*^+/−^, *Cldn11*^*−/−*^ and *Cldn11*^*−/−*^*::Tg*^+/−^ mice at 32 kHz. Wave guidelines in each series highlight the right-shift in wave latency as stimulus intensity decreases for waves I, III, and V. Bold white ‘X’ emphasizes wave V latency at 80 dB SPL for each threshold series. **(B)** Quantified latency/intensity series for *Cldn11*^+/−^*::Tg*^+/−^ and *Cldn11*^*−/−*^*::Tg*^+/−^ mice for ABR wave traces at 32 kHz; 80–40 dB SPL. Peripherally-derived wave I latency-intensity shifts **(B**a) are similar between *Cldn11*^+/−^*::Tg*^+/−^ and *Cldn11*^*−/−*^*::Tg*^+/−^ mice [*F*_(1,20)_ = 0.13, *p* = 0.72]. Centrally-derived waves II **(B**b), III **(B**c) and IV **(B**d) are also comparable [*F*_(1,20)_ = 0.625, *p* = 0.25, *F*_(1,20)_ = 0.14, *p* = 0.71 and *F*_(1,20)_ = 2.36, *p* = 0.14 respectively]; however wave V latency-intensity shifts **(B**e) are significantly increased at each stimulus intensity [*F*_(1,20)_ = 37.3, *p* < 0.0001]. Data plotted as mean ± SEM 10 ≤ *n* ≤ 12; ****p < 0.0001. **(C**,**D)** ABR series at 16 (C) and 8 kHz (D) from the mice in (**A**), are similar to the 32 kHz data, reflecting transgene-mediated rescue of peripheral hearing across much of the frequency spectrum.

To extend our earlier analyses of the Tg(Cldn11)605Gow transgene (Supplementary Figs S1–3), we began by confirming the monaural ABR phenotype of *Cldn11*^*−/−*^*::Tg*^+/−^ mice using 32 kHz pure tone stimuli (Fig. 4A,B). Several hallmark ABR features are apparent. First, five characteristic peaks and troughs are evoked from peripheral and central signal generators^71,72^, beginning with wave I at 1.5–2 ms post stimulus and subsequent waves at approximately 1 ms intervals thereafter (16 and 8 kHz data are similar, Fig. 4C,D). Second, wave latencies are inversely proportional to stimulus intensity. Finally, hearing thresholds of *Cldn11*^+/−^*::Tg*^+/−^ mice and *Cldn11*^*−/−*^*::Tg*^+/−^ mice are indistinguishable (Supplementary Fig. S5), indicating the transgene rescues the peripheral deafness phenotype^42^.

Auditory thresholds notwithstanding, the ABR wave V latency in *Cldn11*^*−/−*^*::Tg*^+/−^ mice is abnormal, with an approximate 1 ms increase at all auditory stimulus intensities (Fig. 4B). In contrast, latencies for waves I – IV are comparable between controls and *Cldn11*^*−/−*^*::Tg*^+/−^ mice, which is expected because the contralateral pathway – from the cochlear nucleus to the LSO – is dominated by large caliber fibers that are only modestly impacted by the absence of claudin 11 (Table 1); 8^th^ nerve fibers are similarly large. For the longest axons in the contralateral pathway (AVCN → MNTB), average waves III – II interpeak latencies interpolated for 60 dB SPL from the latency-intensity regression curves in Fig. 4Bc are 1.06 ± 0.08 ms (*Cldn11*^+/−^*::Tg*^+/−^) and 1.06 ± 0.07 ms (*Cldn11*^*−/−*^*::Tg*^+/−^). These latencies are not statistically different (*t*_*df=20*_, = 0.01, *p* = 0.99); however, we have only 50% power to detect the 70 µs increase in transmission time estimated for *Cldn11*^*−/−*^*::Tg*^+/−^ mice in (Table 1). Despite this limitation, our data preclude several explanations for wave V latency shifts in *Cldn11*^*−/−*^*::Tg*^+/−^ mice such as stimulus artifacts, systemic latency shifts (Supplementary Fig. S5) or degenerative changes in the mutants. Rather, our data are consistent with significantly increased transmission times along small myelinated axons between the AVCN and ipsilateral LSO^43,44,73^.

**Table 1:** Estimated changes in auditory brainstem signal transmission times between the cochlear nucleus (AVCN), medial nucleus of the trapezoid body (MNTB) and lateral superior olive (LSO) in *Cldn11*^+/−^*::Tg*^+/−^ and *Cldn11*^*−/−*^*::Tg*^+/−^ mice.

	AVCN → MNTB^a^	MNTB → LSO^a^	AVCN → LSO^b^
Adult myelinated fiber tract lengths (mm)^89^	4.2	0.92	2.2
Average axon diameters (µm)	3.3	~2^68^	0.92
Est. transmission times, *Cldn11*^+/−^*::Tg*^+/−^ (µs) ^43,44^	280	95	430
Est. transmission times, *Cldn11*^*−/−*^*::Tg*^+/−^ (µs) ^43,44^	350	130	710
**∆ transmission times for** ***Cldn11*** ^*−/−*^ ***::Tg*** ^+/−^ **(µs)**	** + 70**	** + 35**	** + 280**

^a^Contralateral pathway from the AVCN → MNTB → LSO.

^b^Ipsilateral pathway from the AVCN → LSO.

### Abnormal binaural ABRs

In mice, wave V is associated with myelinated principal cells between the SOC and inferior colliculus^73^. The origin of wave V abnormalities in *Cldn11*^*−/−*^*::Tg*^+/−^ mice is likely the SOC because this cluster of nuclei forms a major integration site for signals from the left and right ears^74,75^. Further, our fiber tract analysis shows small and large diameter myelinated fibers converging on the LSO (Fig. 3). Thus, we hypothesized that axon diameter-dependent increases in transmission time along these ipsilateral and contralateral pathways cause abnormal signal processing in the mutants and we examined signal integration and output in greater detail using binaural ABRs associated with interaural level difference measurements at the LSO.

**Figure 5:**
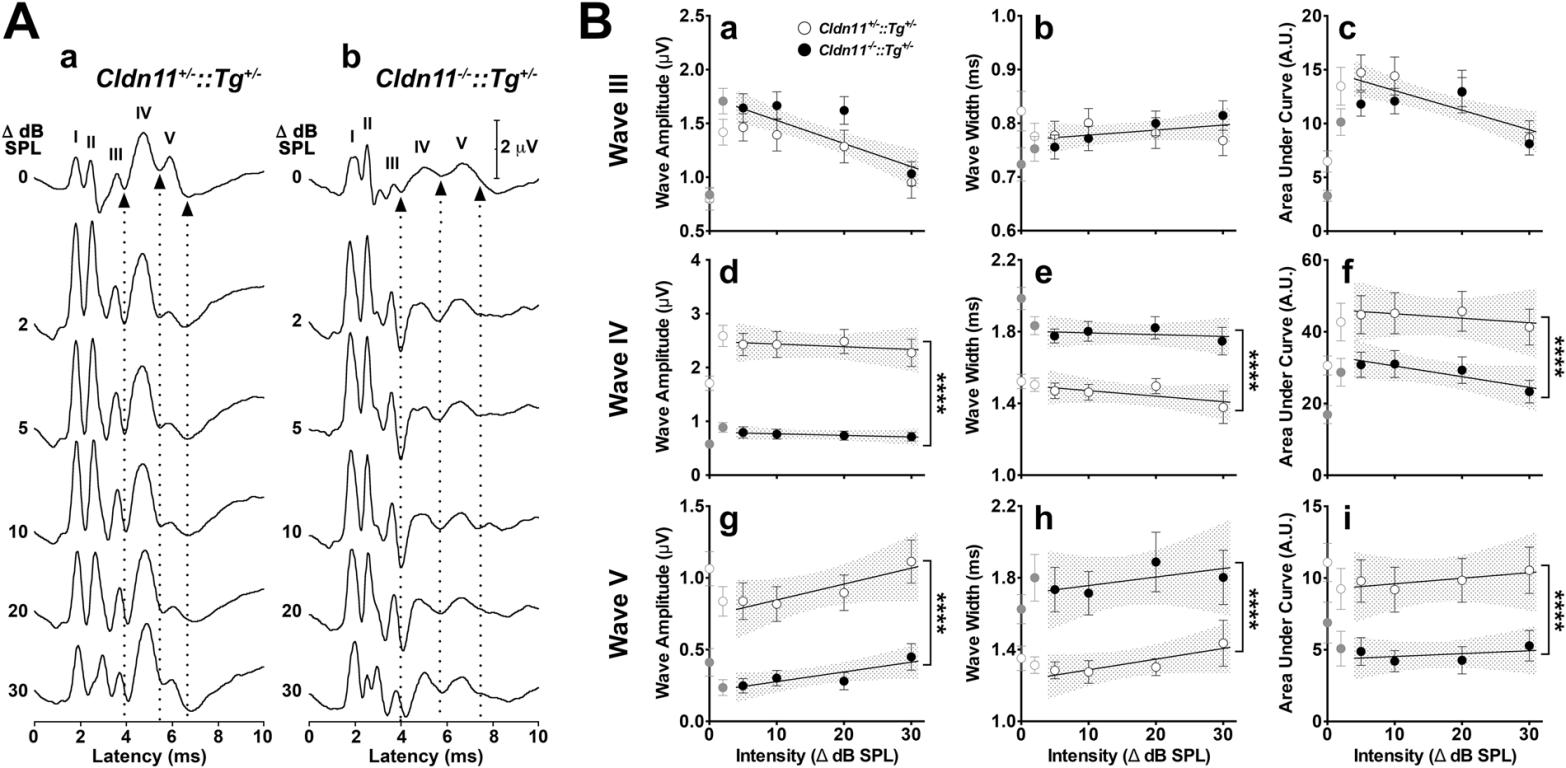
Altered temporal-dispersion of latter binaural ABR waves in *Cldn11*^*−/−*^*::Tg*^+/−^ mice. **(A)** Representative binaural ABR wave graphs from 6–8 week old **(a)** control *Cldn11*^+/−^*::Tg*^+/−^ and (b) *Cldn11*^*−/−*^*::Tg*^+/−^ mice showing changes in ABRs with sound lateralization between 0–30 dB SPL. ABR waves I–V are indicated by roman numerals. Arrowheads and dotted lines show the latencies for the minima of waves III-V (designated waves III’–V’). The widths of waves IV and V appear to be larger for the mutants than the controls, which suggests temporal dispersion. **(B)** Evolution of the characteristics of waves III – V with sound lateralization from 0–30 dB SPL. (a,d,g) Amplitudes for waves III – V measured from each wave maxima to the succeeding III’–V’ troughs. Amplitudes of waves III are comparable for controls versus *Cldn11*^*−/−*^*::Tg*^+/−^ mice as sound is lateralized [*F*_(2,83)_ = 2.59, *p* = 0.08], but we observe significant decreases for waves IV [*F*_(2,83)_ = 107.6, *p* < 0.0001] and V [*F*_(2,82)_ = 38.4, *p* < 0.0001]. (b,e,h) Widths of waves III – V measured between the preceding and succeeding wave trough minima. Widths of waves III are consistent between genotypes [*F*_(2,83)_ = 1.42, *p* < 0.25], but there are significant increases for waves IV [*F*_(2,83)_ = 32.9, *p* < 0.0001] and V [*F*_(2,82)_ = 15.5, *p* < 0.0001]. (c,f,i) Area under the curve for waves III–V measured with baselines drawn between the preceding and succeeding troughs. Areas are indistinguishable for waves III [*F*_(2,80)_ = 1.47, *p* = 0.24] but are increased for waves IV [*F*_(2,80)_ = 12.8, *p* < 0.0001] and V [*F*_(2,80)_ = 17.9, *p* < 0.0001]. Data points from 5–30 dB SPL intensity differences analyzed using linear regression least-squares fits to determine if the slopes and *Y*-intercepts differ between control (*n* = 10) and mutant (*n* = 11–12) mice. Stippled regions reflect 95% confidence intervals for the linear regression fits. Slopes and *Y*-intercepts are indistinguishable between genotypes for all wave III characteristics [*F*_(2,80–82)_; *p* > 0.08]. Slopes and *Y*-intercepts are not shared between genotypes for any characteristics of waves IV or V [*F*_(2,80–83)_; *p* < 0.0001]. The *Y*-intercepts show lower amplitudes for the knockouts (wave IV = 32%; wave V = 28%), increased wave widths (wave IV = 120%; wave V = 139%) and reduced areas under the curve (wave IV = 72%; wave V = 47%). Data in (B) plotted as mean ± SEM.

The morphology of binaural ABRs are similar for each genotype and similar to monaural ABRs, although relative wave amplitudes differ and the latencies of all waves are increased by approximately 500 µs (Fig. 5A). However, detailed analysis of waves IV and V from *Cldn11*^*−/−*^*::Tg*^+/−^ mice reveals several abnormalities. First, the amplitudes are significantly lower than controls (Fig. 5Bd,g), suggesting temporal dispersion in the LSO. Second, the widths are increased (Fig. 5Be,h), which indicates greater temporal dispersion (loss of synchrony) of action potentials. Finally, the areas under waves IV and V are reduced (Fig. 5Bf,i), which suggests overall diminished recruitment of LSO neurons, despite normal activity at the MNTB (Fig. 5Ba–c) wherein wave III amplitudes, widths and areas are comparable to controls. Together, these data indicate that binaural ABRs are superior to monaural ABRs for detecting central auditory deficits and that the SOC and associated ABR waves IV and V are abnormal in *Cldn11*^*−/−*^*::Tg*^+/−^ mice.

### Sound lateralization deficit

**Figure 6:**
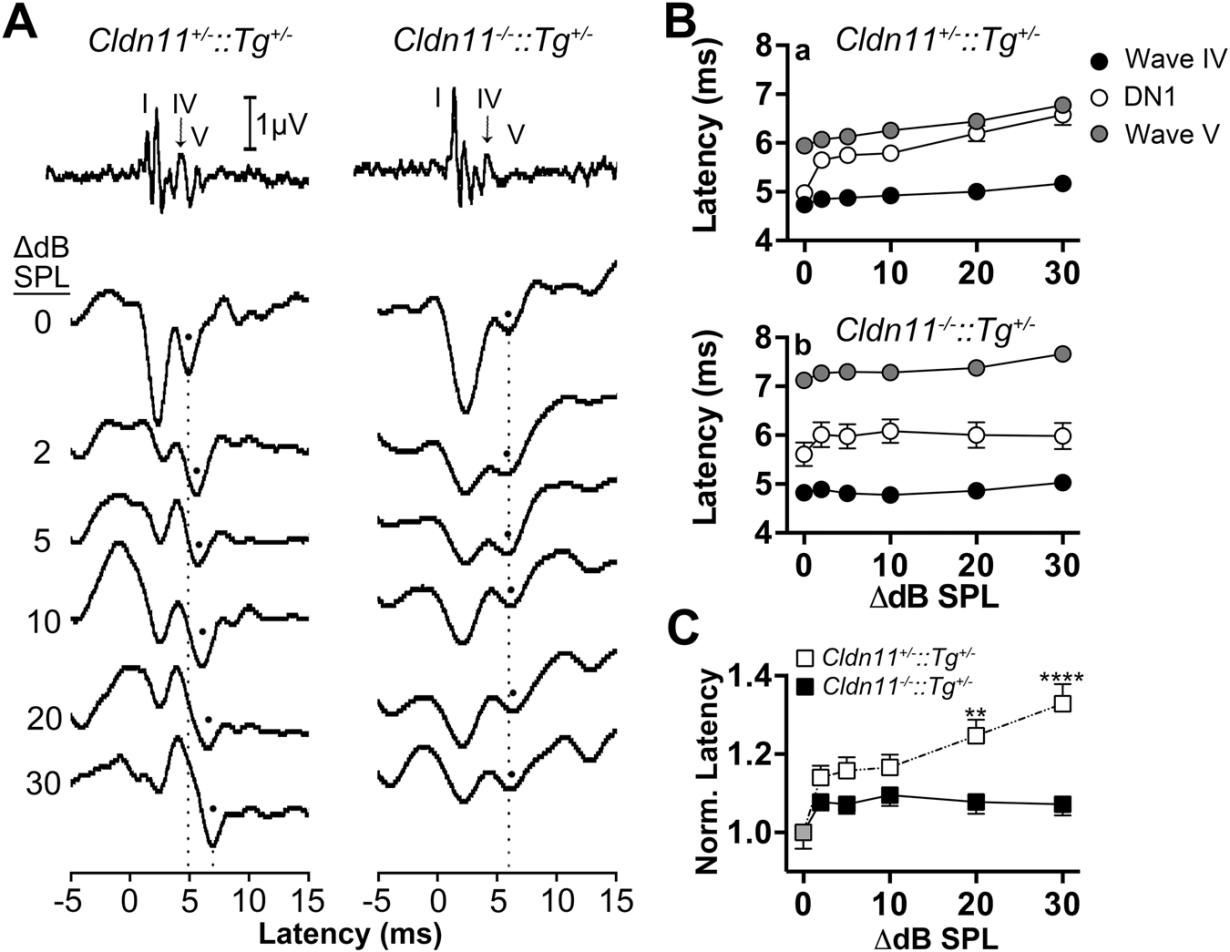
*Cldn11*^*−/−*^*::Tg*^+/−^ mice exhibit abnormal sound lateralization. **(A)** Representative binaural interaction component (BIC) traces from *Cldn11*^+/−^*::Tg*^+/−^ and *Cldn11*^*−/−*^*::Tg*^+/−^ mice calculated for stimulus intensity differences between each ear of 0–30 dB SPL. Black dots identify the processed binaural output from those intensity differences, which are the DN1 troughs. Monaural ABR traces (top) are in temporal alignment with the BICs to highlight the latency of the processed binaural DN1 trough, which occurs between monaural evoked latencies of waves IV and V. Dashed lines highlight the latency of DN1 for *Cldn11*^+/−^*::Tg*^+/−^ mice as sound is lateralized (Δ0 to Δ30 dB SPL respectively) and draw attention to the lack of shift in *Cldn11*^*−/−*^*::Tg*^+/−^ mice. **(B)** Latency/intensity series comparing latency of ABR waves IV (black circles) and V (grey circles) to the calculated DN1 trough (white circles) for **(B**a) *Cldn11*^+/−^*::Tg*^+/−^ and **(B**b) *Cldn11*^*−/−*^*::Tg*^+/−^ mice. Note the pathological increased wave V latency for *Cldn11*^*−/−*^*::Tg*^+/−^ mice. **(C)** Comparison of DN1 trough latencies for *Cldn11*^+/−^*::Tg*^+/−^ and *Cldn11*^*−/−*^*::Tg*^+/−^ mice. Lateralized DN1 trough latencies (Δ2–30 dB SPL) were normalized to the midline response latency (Δ0 dB SPL) for each animal. As sound is lateralized, the DN1 interaction trough latency is significantly different between *Cldn11*^+/−^*::Tg*^+/−^ and *Cldn11*^*−/−*^*::Tg*^+/−^ mice [*F*_(1,20)_ = 10.3, *p* = 0.005]. Grey circles represent the normalized value for both *Cldn11*^+/−^*::Tg*^+/−^ and *Cldn11*^*−/−*^*::Tg*^+/−^ mice. Data plotted as mean ± SEM; 10 ≤ *n* ≤ 12; **p < 0.01, ****p < 0.0001.

The deficits we observe in LSO neurophysiology from *Cldn11*^*−/−*^*::Tg*^+/−^ mice rouse expectations that a major function of this nucleus is compromised. To investigate further, we derived binaural interaction components (BICs) from the binaural ABRs between waves IV and V, which reflect signal processing in the LSO. Overall, BICs for control and *Cldn11*^*−/−*^*::Tg*^+/−^ mice (Fig. 6A) are similar. Two major troughs are present in all ∆ dB SPL traces, the second of which is between the latencies of ABR waves IV and V and is designated the DN1 peak (Fig. 6B).

Nevertheless, there are several differences. First, DN1 latencies are larger for the mutants (Fig. 6B) which is consistent with ABR wave V latencies (Figs 4 and 5). Second, DN1 latencies in controls are proportional to stimulus intensity differences between the two ears as previously reported^51,76^, while the mutant DN1 latencies are independent of ∆ dB SPL (Fig. 6C). The final difference is the larger amplitudes for the controls, measured from DN1 minima to DN1’ maxima.

In similar vein to the smaller areas under binaural ABR waves IV and V from *Cldn11*^*−/−*^*::Tg*^+/−^ mice, we would expect smaller DN1 amplitudes; unfortunately, the amplitudes are unexpectedly variable for both genotypes and the data yield no interpretable results. Our analysis of DN1 latencies is consistent with previous reports^77,78^ and indicate that lateralizing sound sources in wild type mice increases BIC latencies. This does not occur in *Cldn11*^*−/−*^*::Tg*^+/−^ mice, which reveals an abnormality that would likely distort perception of the auditory field and perturb behavioral responses to stimuli.

### Altered neurochemistry in the SOC

**Figure 7:**
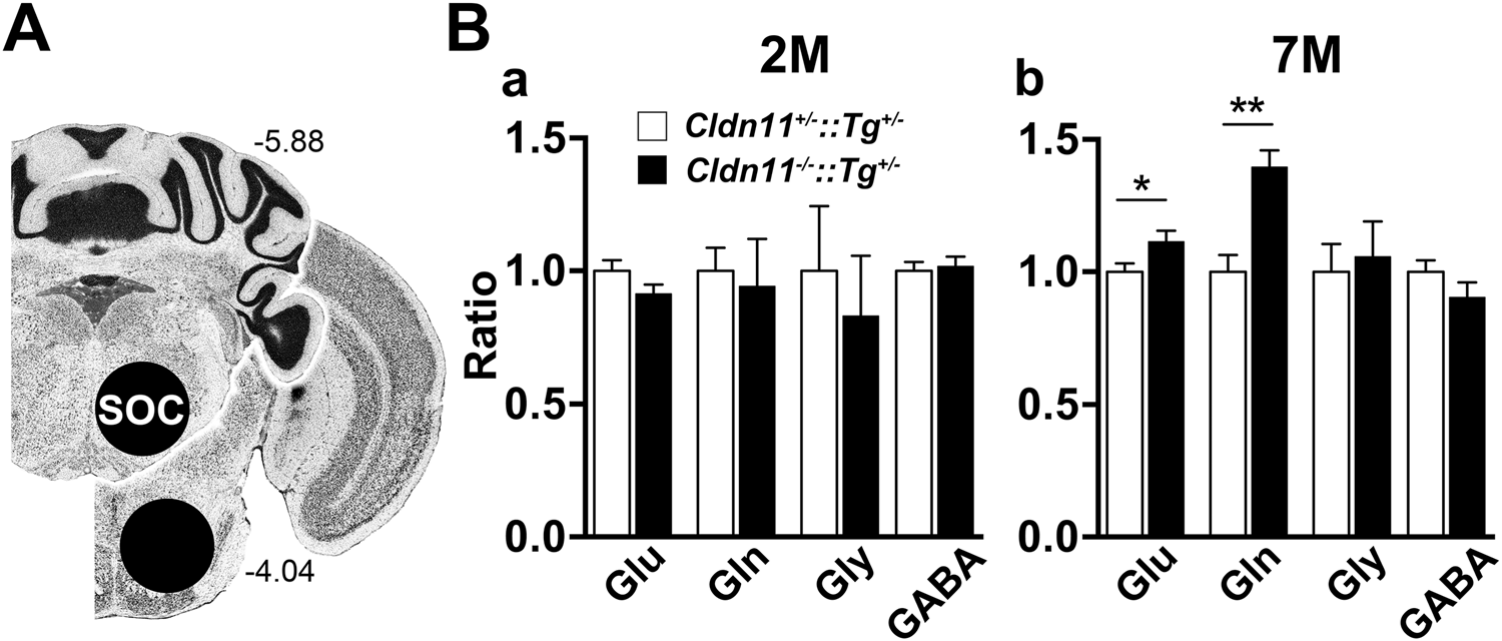
^1^H-MRS analysis of the SOC indicates altered neurotransmitter levels in *Cldn11*^*−/−*^*::Tg*^+/−^ mice. **(A)** Schematic demarcating the rostral/caudal Bregma boundaries of brain slices and SOC tissue punch locations for *ex vivo* neurochemistry analysis using magic-angle ^1^H-MRS at 11.7 Tesla. **(B)** Select metabolite analysis in *Cldn11*^+/−^*::Tg*^+/−^ and *Cldn11*^*−/−*^*::Tg*^+/−^ mice. (**B**a) Relevant inhibitory and excitatory neurotransmitter levels normalized to creatine in 2 month old (2 M) SOC. No changes in neurotransmitter level are observed for glutamate [*t*_(9)_ = 1.58, *p* = 0.15], glutamine [*t*_(9)_ = 0.308, *p* = 0.77], glycine [*t*_(9)_ = 0.512, *p* = 0.63], or GABA [*t*_(9)_ = 0.334, *p* = 0.75]. (**B**b) Relevant inhibitory and excitatory neurotransmitter levels normalized to creatine in 7 M SOC. Significant increases in glutamate [*t*_(9)_ = 2.24, *p* = 0.048] and its precursor glutamine [*t*_(9)_ = 4.40, *p* = 0.0013] were detected, but inhibitory neurotransmitter levels remain similar; glycine: *t*_(9)_ = 0.332, *p* = 0.747, GABA: *t*_(9)_ = 1.33, *p* = 0.21. Neurochemical levels for each mouse are internally normalized to creatine and *Cldn11*^*−/−*^*::Tg*^+/−^ values are expressed relative to *Cldn11*^+/−^*::Tg*^+/−^ and plotted as mean ± SEM; *n* = 6; *p < 0.05, **p < 0.01. Panel A: Image Credit, Allen Institute.

In light of this presumptive behavioral phenotype, we used ^1^H-MRS to examine auditory brainstem neurochemistry and determine if the increased transmission time^41–44^ of ipsilateral fibers might also be associated with altered neurotransmitter levels. Brain slice stereotactic coordinates show the locations of bilateral tissue punches analyzed from 2 and 7 month old (2 and 7 M) *Cldn11*^+/−^*::Tg*^+/−^ and *Cldn11*^*−/−*^*::Tg*^+/−^ mice (Fig. 7A). Neurotransmitters in the adult auditory brainstem are dominated by glutamate and glycine, although several published studies also report the presence of GABA. At 2 M, we observe no differences in these neurotransmitter levels, as determined from areas under the relevant proton spectral peaks (Fig. 7B). We also find no differences in 16 other neurochemicals including N-acetylaspartate (see Methods for list), which is a biomarker for neuron damage.

At 7 M, we also observe no differences in glycine or GABA between the genotypes. In contrast, glutamate levels are increased in *Cldn11*^*−/−*^*::Tg*^+/−^ mice by more than 10% compared to controls, and its metabolic precursor glutamine is increased 1.4-fold. Succinate levels are normal in these mice, suggesting that TCA cycle-derived α-ketoglutarate does not contribute to increased glutamate and glutamine levels in the mutants, which we conclude more likely signify elevated excitatory tone from glutamatergic neurons synapsing onto SOC nuclei, or from SOC neurons themselves. We hypothesize that modulating excitatory tone partly offsets the apparent reduction in principal cell recruitment in the mutants (Fig. 5B), which is consistent with a compensatory mechanism for diminished SOC output (persistent from an early age; Figs 4–6) because glutamate and glutamine levels are increased at 7 M but not 2 M.

### Additional affective behavioral dysfunction

Our early hypothesis that conduction velocity slowing along small myelinated fibers could alter behavior is supported anecdotally by our observation that *Cldn11*^*−/−*^*::Tg*^+/−^ mice often react abnormally by lingering in the center of home cage when the filter top is removed, while littermate controls scatter to the edges (thigmotaxis). This observation suggests diminished anxiety-like or avoidance behavior, which we quantified using open field (OF) and marble burying (MB) tests at 2 and 7 M to determine age of onset.

**Figure 8:**
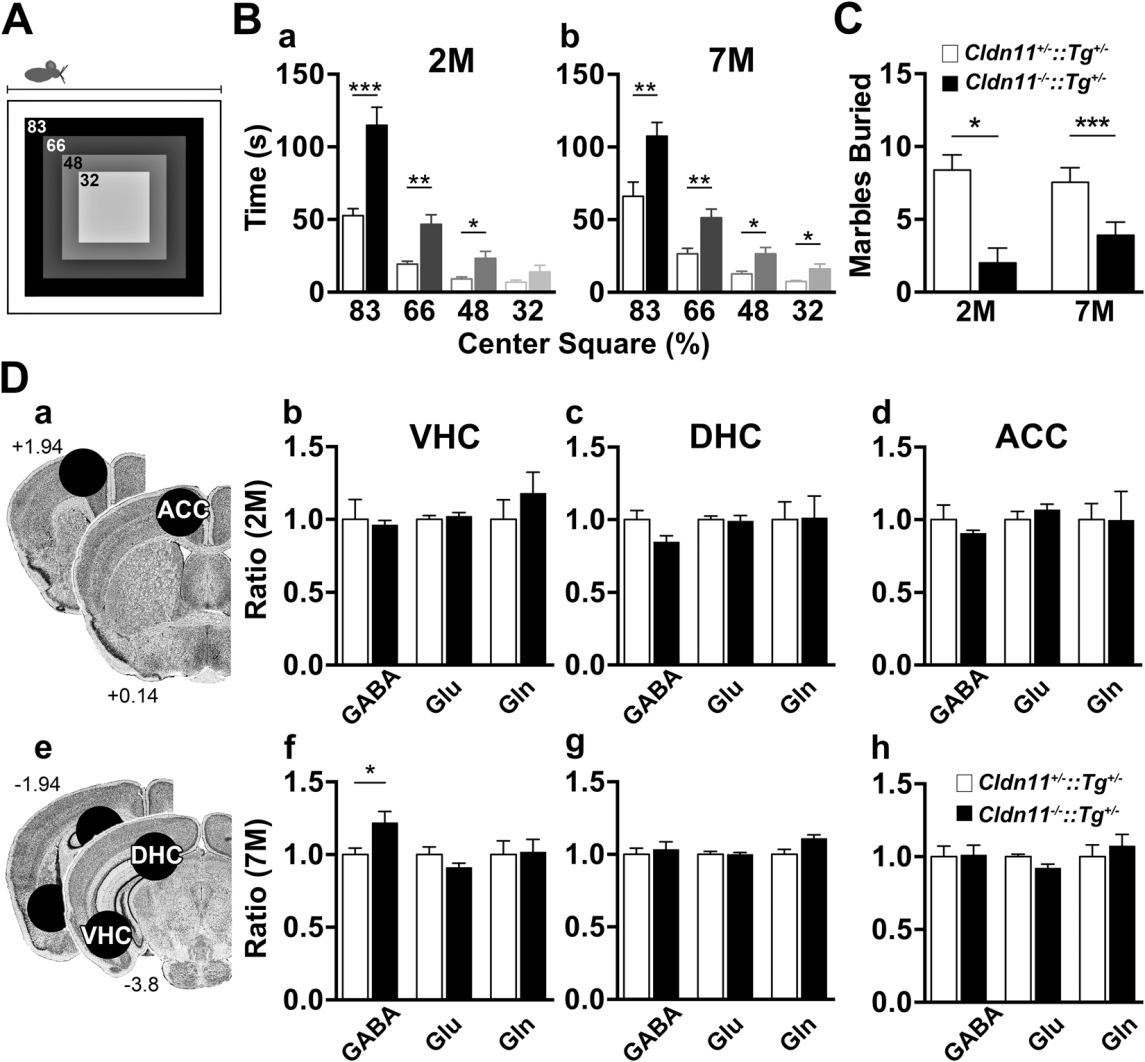
*Cldn11*^-/–^*::Tg*^+/−^ mice have a decreased anxiety-like endophenotype. **(A)** Schematic depicting the open field (OF) arena and four virtual concentric center squares. **(B)** Analyses for each virtual center square for *Cldn11*^+/−^*::Tg*^+/−^ and *Cldn11*^*−/−*^*::Tg*^+/−^ mice at (**B**a) 2 month old (2 M) and (**B**b) 7 M. At both ages, *Cldn11*^*−/−*^*::Tg*^+/−^ mice spend significantly more time near the center of the arena compared to controls. At 2 M: 83%, *t*_(17)_ = 4.50, *p* = 0.0003; 63%, *t*_(17)_ = 3.78, *p* = 0.0015; 48%, *t*_(17)_ = 2.66, *p* = 0.017; 32%, *t*_(17)_ = 1.41, *p* = 0.18. At 7 M: 83%, *t*_(18)_ = 3.07 *p* = 0.007; 63%, *t*_(18)_ = 3.35, *p* = 0.004; 48%, *t*_(18)_ = 2.75, *p* = 0.014; 32%, *t*_(18)_ = 2.49, *p* = 0.024. **(C)** Marble burying (MB) analyses for *Cldn11*^+/−^*::Tg*^+/−^ and *Cldn11*^*−/−*^*::Tg*^+/−^ mice at 2 and 7 M. The *Cldn11*^*−/−*^*::Tg*^+/−^ mice bury significantly fewer marbles than controls at both ages [2 M, *t*_(17)_ = 2.72, *p* = 0.015; 7 M, *t*_(18)_ = 4.40, *p* = 0.0003], which corroborates the reduced anxiety phenotype. **(D)** Analysis of key brain regions relevant to anxiety state for *Cldn11*^+/−^*::Tg*^+/−^ and *Cldn11*^*−/−*^*::Tg*^+/−^ mice. (**D**a,e) Schematics delineate rostral and caudal stereotaxic boundaries (with respect to Bregma) and tissue punch locations for *ex vivo* analysis of 19 neurochemicals (normalized to creatine levels) using ^1^H-MRS at 500 MHz (11.7 Tesla). (**D**b–d) Relative inhibitory and excitatory neurotransmitter levels measured at 2 M in amygdala/ventral hippocampus (VHC) (b), dorsal hippocampus (DHC) (c) and anterior cingulate cortex (ACC) (d). Similar levels of all neurotransmitters are observed between *Cldn11*^+/−^*::Tg*^+/−^ and *Cldn11*^*−/−*^*::Tg*^+/−^ mice [VHC: GABA, *t*_(10)_ = 0.27, *p* = 0.79; Glu, *t*_(10)_ = 0.54, *p* = 0.60, Gln, *t*_(10)_ = 0.90, *p* = 0.39. DHC: GABA, *t*_(10)_ = 2.10, *p* = 0.065; Glu: *t*_(10)_ = 0.24, *p* = 0.82; Gln: *t*_(10)_ = 0.06, *p* = 0.96. ACC: GABA, *t*_(10)_ = 0.33, *p* = 0.75; Glu, *t*_(10)_ = 1.58, *p* = 0.15; Gln, *t*_(10)_ = 0.31, *p* = 0.77]. **(D**f–h) Relative inhibitory and excitatory neurotransmitter levels measured at 7 M in VHC (b), DHC (c) and ACC (d). GABA levels are significantly increased in VHC of *Cldn11*^*−/−*^*::Tg*^+/−^ mice [*t*_(9)_ = 1.50, *p* = 0.034] but normal in DHC [*t*_(9)_ = 0.49, *p* = 0.64] and ACC [*t*_(9)_ = 0.112, *p* = 0.91]. Excitatory neurotransmitters are comparable to controls [VHC: Glu, *t*_(9)_ = 1.36, *p* = 0.21; Gln, *t*_(9)_ = 0.132, *p* = 0.90. DHC: Glu, *t*_(9)_ = 0.037, *p* = 0.97; Gln, *t*_(9)_ = 2.43, *p* = 0.038. ACC: Glu, *t*_(9)_ = 2.30, *p* = 0.05; Gln, *t*_(9)_ = 0.630, *p* = 0.54]. Neurochemical levels for each mouse are internally normalized to creatine and *Cldn11*^*−/−*^*::Tg*^+/−^ values are expressed relative to *Cldn11*^+/−^*::Tg*^+/−^ and plotted as mean ± SEM; 5 ≤ *n* ≤ 6; *p < 0.05, **p < 0.01. Panels Da and De: Image Credit, Allen Institute.

An OF arena schematic (Fig. 8A) shows the four virtual concentric center squares used for analysis. At 2-months (2 M) of age (Fig. 8Ba), *Cldn11*^*−/−*^*::Tg*^+/−^ mice spend significantly more time than controls in all center squares except the 32% center square. The *Cldn11*^*−/−*^*::Tg*^+/−^ mice travel greater distances with increased velocity, which indicates they are not immobile during testing but, more likely, exhibit a reduced anxiety phenotype (Supplementary Fig. S6). The lack of preference for adjacent quadrants of the arena indicates minimal influence on behavior by environmental cues.

The reduced anxiety phenotype persists until at least 7 M where, again, *Cldn11*^*−/−*^*::Tg*^+/−^ mice spend more time in all center squares of the arena (Fig. 8Bb) while traveling similar distances at comparable velocity to controls, and with similar quadrant bias (Supplementary Fig. S6). The MB tests were performed on the same cohorts of mice and corroborate the reduced anxiety phenotype; thus at both 2 and 7 M, *Cldn11*^*−/−*^*::Tg*^+/−^ mice bury significantly fewer marbles than controls (Fig. 8C).

Behavioral abnormalities, including anxiety and depression, are frequently comorbid with white matter diseases such as multiple sclerosis and leukodystrophy^79^. In light of altered anxiety inferred by the OF and MB tests, we sought to determine if the behavioral abnormalities of *Cldn11*^*−/−*^*::Tg*^+/−^ mice also include a depression-like endophenotype. Thus, we examined these mutants using tail suspension tests (TST) at 2 or 7 M (Supplementary Fig. S6). At both ages there are no significant differences between control and *Cldn11*^*−/−*^*::Tg*^+/−^ mice. Together, these data indicate that *Cldn11*^*−/−*^*::Tg*^+/−^ mice exhibit a reduced anxiety/avoidance-like behavior in the absence of a depression-like endophenotype.

### Altered amygdala neurochemistry

The reduced anxiety phenotype in *Cldn11*^*−/−*^*::Tg*^+/−^ mice is apparent from an early age and persists for at least 7 M, demonstrating that the absence of claudin 11^43,44^ causes overt and long lived behavioral phenotypes. To determine if white matter dysfunction alters neurochemistry in anxiety-associated circuits, we performed proton magnetic resonance spectroscopy (^1^H-MRS) on *ex vivo* tissue punches from three brain regions, ventral and dorsal hippocampus and anterior cingulate cortex (VHC, DHC, ACC; Fig. 8Da,e) from the OF/MB/TST mouse cohorts.

At 2 M, levels of the inhibitory neurotransmitter GABA and the excitatory neurotransmitter glutamate, as well as its precursor glutamine, are normal in *Cldn11*^*−/−*^*::Tg*^+/−^ mice compared to controls (Fig. 8Db–d. In addition, levels of 16 other neurochemicals detectable by ^1^H-MRS are normal (see Methods for list), including N-acetylaspartate which indicates that neurodegeneration is negligible in the mutants. These data indicate that significant behavioral phenotypes may arise in the absence of detectable neurotransmitter changes or neuron damage, and are presumably driven by temporal disconnection of sensory information disseminated by myelinated axons between integration and processing nuclei.

By 7 M, GABA is increased by more than 20% in the amygdala/VHC of *Cldn11*^*−/−*^*::Tg*^+/−^ mice (Fig. 8Df), while levels in upstream (ACC) and downstream (DHC) nuclei are comparable to controls (Fig. 8Dg,h). In contrast, glutamate and glutamine levels are indistinguishable from controls (Fig. 8Df–h). The major neurotransmitter system used by neurons for amygdala and VHC connections is GABA; thus, these data reflect a change in neurochemistry that is site-specific and consistent with the reduced anxiety phenotype of *Cldn11*^*−/−*^*::Tg*^+/−^ mice. Again, N-acetylaspartate levels are normal.

## Discussion

In the current study, we tackle an heretofore intractable problem in behavioral neuroscience – that of characterizing molecular links between dysfunctional myelin and behavioral changes in the absence of degenerative pathology – and demonstrate how and why disrupting myelin function should be considered a plausible etiology for psychiatric disease alongside other well-characterized neuron-specific etiologies. Addressing this issue has been possible only because the mechanism of myelin tight junction function is known^41,43–45^, and the myelin dysfunction in *Cldn11*^*−/−*^*::Tg*^+/−^ mice does not lead to neurodegeneration. Thus, the electrical resistance of CNS myelin is decreased, particularly in small diameter fibers, which increases transmission time and may temporally disconnect processing/integration nuclei from distributed brain networks.

The *Cldn11*^*−/−*^*::Tg*^+/−^ mice exhibit a reduced anxiety/avoidance phenotype from early adulthood in the absence of a depression-like endophenotype and eventually develop a neurotransmitter imbalance whereby steady state GABA levels in the amygdala/ventral hippocampus rise more than 20%. The magnitude of increase is similar to those in patients with anxiety disorder^80–82^, and twice that observed in other rodent anxiety models^83,84^. Thus, our data lead inexorably to expansion of possible etiologies underlying psychiatric disease.

Direct demonstration of the links between myelin dysfunction, abnormal behavior and neurotransmitter imbalance are technically challenging in the limbic system because of the broad and complex distribution of incumbent nuclei and the absence of suitable non-invasive techniques with which to identify changes in transmission time. However the auditory pathway is physically-restricted, exquisitely interwoven for temporal and spatial processing of sensory information and perhaps uniquely amenable to interrogation using sophisticated neurophysiologic techniques.

From the youngest to the oldest ages that we can consistently record auditory responses, monaural ABRs from *Cldn11*^*−/−*^*::Tg*^+/−^ mice are normal overall; although, wave V latency is increased at all stimulus intensities and pure tone frequencies spanning two octaves. Binaural ABRs are more sensitive than monaural measurements, and can reveal subtle abnormalities in signal amplitude/dispersion or neuron recruitment for the major nuclei of the superior olivary complex. Indeed, we demonstrate functional changes in the lateral superior olivary nucleus, important functions of which include processing/integration of auditory signals from left and right ears according to stimulus intensity differences and transmission of this information to inferior colliculus^74,75,85^. In contrast to controls, the latency of this output is invariant in *Cldn11*^*−/−*^*::Tg*^+/−^ mice, demonstrating at least partial loss of sensory information pertaining to sound location in the azimuth plane. Such a deficit could be associated with a behavioral phenotype in freely behaving *Cldn11*^*−/−*^*::Tg*^+/−^ mice by triggering inappropriate responses to auditory stimuli.

Consistent with our hypothesis, we also show that the absence of claudin 11 in myelin disrupts LSO function in *Cldn11*^*−/−*^*::Tg*^+/−^ mice, which in large part could reflect temporal dispersion between the AVCN and the ipsilateral LSO. These nuclei are connected via small myelinated axons averaging 0.9 µm in diameter, and our previous modeling experiments suggest conduction velocity along these fibers could drop to <60% of normal^43,44^. In adult wild type mice, the transmission time for these axons approximates 430 µs, which we estimate could increase by 280 µs in *Cldn11*^*−/−*^*::Tg*^+/−^ mice (Table 1). Myelinated contralateral efferents from the AVCN are >3 µm in diameter and the estimated transmission time of 280 µs should increase by only 70 µs in the absence of claudin 11. Thus, our estimates indicate that signals transiting the ipsilateral pathway are increased to a greater extent than those in the contralateral pathway.

Efferents from the MNTB are glycinergic and hyperpolarize LSO principal neurons. In contrast, LSO afferents from the ipsilateral AVCN are excitatory and counterbalance the contralateral signals, thereby modulating the amplitude and temporal features of signals to inferior colliculus. In *Cldn11*^*−/−*^*::Tg*^+/−^ mice, disproportionate increases in transmission time along the ipsilateral axons likely disperse LSO output, leading to smaller and broader signals, which we observe for waves IV and V (Fig. 5B). From the perspective of neurochemistry, the higher steady state levels of glutamate and glutamine that we observe in older mutants (Fig. 7B) suggest that persistent signal temporal dispersion in the LSO may gradually increase firing of glutamatergic spherical bushy cells in the ipsilateral AVCN as a compensatory mechanism to boost signals for subsequent auditory nuclei up to the cortex and potentially beyond.

Together our data reveal two behavioral phenotypes, associated with altered regulation of inhibitory and excitatory neurotransmitters in distinct brain regions, all caused by a monogenic loss-of-function defect. Further, it is tempting to speculate that additional integration circuits relying on large and small myelinated afferents could be altered in the mutants. If so, it is also reasonable to expect that the homeostasis of additional neurotransmitter systems in those circuits eventually might be perturbed, leading to a multimodal behavioral phenotype affecting several circuits in different brain regions.

While the anxiety phenotype of *Cldn11*^*−/−*^*::Tg*^+/−^ mice is readily interpreted as an increased risk of predation in Darwinian settings, diminished proficiency for localizing sound in space may seem more abstract. However, auditory field distortions stemming from inaccurate sound source localization may have untoward consequences for predator detection and evasion. And although not privy to the precepts of *Cldn11*^*−/−*^*::Tg*^+/−^ mice, we can imagine situations where a multimodal phenotype might emerge as a unified behavioral deficit; for example, a general ambivalence to danger or a lack of awareness escalating to apparent impulsive or risk-taking behavior should a mouse attempt escape toward a predator (rather than the opposite direction) in response to auditory startle.

Despite a mechanistic analysis of the auditory pathway in *Cldn11*^*−/−*^*::Tg*^+/−^ mice, our assertion that myelin dysfunction can cause behavioral phenotypes would be strengthened by corroborating clinical data. But in lieu of null alleles of the *CLDN11* gene in patients, we rely on precedence from auditory brainstem lesions in infarct and MS patients^86,87^. Interaural level difference testing for these patients, which is analogous to sound lateralization in the current study, revealed profound distortions of the auditory field in 12/14 patients with lesions in the SOC, the inferior colliculus or the connecting myelinated fiber tracts between these two nuclei. Thus, all sound presentations emanating from locations across the azimuth plane were perceived by the patients either to come from the right, or left, or center of the auditory field. Similar to mice, the human auditory pathway from the SOC to the inferior colliculus gives rise to ABR waves IV and V and these clinical data lend strong support to our interpretation of the neurophysiology from *Cldn11*^*−/−*^*::Tg*^+/−^ mice.

**Figure 9:**
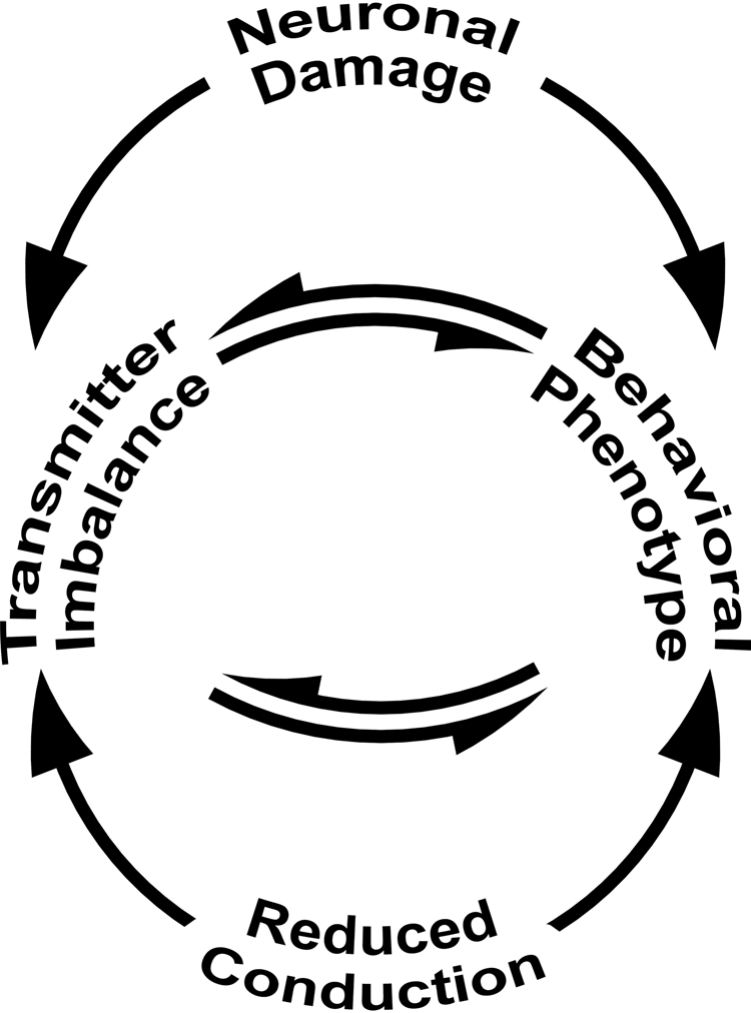
Mechanisms of altered behavior and neurochemistry. The disconnection hypothesis^1^ highlights the importance of temporally-coordinated sensory information transfer and processing in spatially-distributed brain circuits for normal behavior. In this framework, psychiatric disease is proposed to arise from the disconnection of different brain regions, and well-known mechanisms include abnormal neuronal targeting during development, loss of cortical or other types of neurons and neurotransmitter imbalances. The schematic includes an additional mechanism by which neural circuits can be disconnected, embodied by the disconnection syndrome^32,58^. This mechanism is demonstrated for two brain regions in the current study, whereby altered transmission time along myelinated axons disrupts communication within spatially distributed circuits. We demonstrate that different neurotransmitter systems are impacted in the different brain regions and that temporal disconnection, even in the absence of degeneration, causes behavioral changes and neurotransmitter imbalance.

In this light, we conclude that dysfunctional myelin is appropriately considered as a potential etiology for psychiatric disease in some patients (Fig. 9). Herein, we demonstrate in the limbic system and the auditory pathway, that changes to the passive properties of myelin cause early behavioral abnormalities of diminished anxiety- or avoidance-like behavior and distortion of the auditory field. We attribute the eventual changes in neurotransmitter homeostasis to compensatory mechanisms that modulate output in neural circuits, possibly operating via feedback and feed forward loops. Importantly, the monogenic defect in *Cldn11*^*−/−*^*::Tg*^+/−^ mice potentially causes a complex multimodal phenotype involving at least two neurotransmitter systems which, in humans, might not be amenable to simple monotherapies for effective symptom management. Future work will focus on awake behaving *Cldn11*^*−/−*^*::Tg*^+/−^ mice to determine if they can localize sounds in space and to identify small molecules that ameliorate the behavioral phenotypes.

## Electronic supplementary material

Supplementary Methods and Figures
