# Supplementary material for: Absence of Claudin 11 in CNS Myelin Perturbs Behavior and Neurotransmitter Levels in Mice
Source: Sci Rep. 2018 Feb 28;8:3798. doi: 10.1038/s41598-018-22047-9 (PMC5830493; doi:10.1038/s41598-018-22047-9)
Supplement: Supplementary file 1 — Supplementary Methods and Figures [file 41598_2018_22047_MOESM1_ESM.pdf]

Absence of Claudin 11 in CNS Myelin Perturbs Behavior and Neurotransmitter Levels in Mice

Maheras KJ, Peppi M, Ghoddoussi F, Galloway MP, Perrine SA, Gow A

Supplementary Methods

Auditory fiber tract tracing

Auditory fiber tract tracing was accomplished by modifying published procedures [1,2]. Mice were perfused with 10ml of freshly oxygenated artificial cerebral spinal fluid (aCSF: 126mM NaCl, 3mM KCl, 2mM CaCl2, 2mM MgSO4, 1.25mM NaH2 PO4, 26mM NaHCO3, 10mM dextrose, pH7.4) at a rate of 2ml/min. Brains were promptly removed and 2mm coronal sections isolating the lower auditory brainstem nuclei were harvested ranging from interaural -1 to -3 using a mouse anodized aluminum matrix (Braintree Scientific, Braintree, MA). Sections were allowed to rest for 1hr in a 60 X 60mm petri dish filled with continuously oxygenating aCSF.

Custom microinjection needles 10–20µm in diameter were pulled from glass capillary tubes using a glass electrode puller (Narishige International Inc., East Meadow, NY) and backfilled with 3µl of a 50mg/ml solution containing Alexa488 labeled Dextran (Molecular Probes, Eugene OR). Needles were attached onto a digital micro-pipette (VWR International, Radnor, PA) fastened to a micromanipulator (Narishige International Inc., East Meadow, NY).

Auditory brainstem slices were transferred from the resting dish into a separate 60mm dish containing freshly carbox-perfused aCSF under a stereomicroscope (Leica, Solms, Germany). The tip of the needle was inserted 0.2 – 0.8mm below the surface of the tissue sections and 30nl of dye injected into target nuclei or fiber tracts. Dye was injected unilaterally in 1 – 3 bolus deposits and either MNTB or LSO connections were analyzed in each tissue slice. Dextran-labeled fibers from cochlea nucleus injections that entered the contralateral MNTB and were analyzed, as were ipsilateral dextran-labeled fibers entering the LSO. Brain slices were perfused with oxygenated aCSF for 4–6hr to allow for dye uptake at the site of injection and transport down the axons. Slices were fixed overnight with fresh 4% paraformaldehyde (PFA; Sigma, St Louis, MO) at 4°C with gentle rotation then washed in PBS for vibratome sectioning.

Dextran dye-labeled coronal mouse brain sections were removed from PBS, blotted dry, placed into a 15 x 15mm cryomold (Electron Microscopy Science, Thermo Fisher) and overlaid with 4% low-melt agarose (Agarose Products, Hernando, MS). Once solidified, molds were removed, the gel excised, trimmed, and superglued to a vibratome chuck. Coronal sections 70µ m thick were cut (Vibratome Series 1000 Plus) and transferred to a 24-well dish (Thermo Fisher) for antibody labeling.

Individual 70µm vibratome sections were stained in 24-well dishes. Sections were permeabilized for 1hr in blocking solution: 0.5% triton X-100 in TBSGBA (1X TBS pH7.5, 0.1% gelatin (Sigma), 1% BSA, 0.05% sodium azide, 2% goat serum (Gibco, Thermo Fisher). Anti-Calbindin D28K antibody (Sigma) in blocking solution was incubated for three days with gentle agitation at 4°C.

Slices washed 2 x 30min with 0.5% triton X-100 in PBS. Secondary antibodies conjugated with Alexa568 (Molecular Probes) were diluted in blocking solution and added to the sections for 6hr at room temperature with gentle agitation. The secondary antibodies were removed and replaced with a DAPI solution (Sigma) diluted in PBS plus 0.5% triton X-100 for 10min. Finally, sections were washed 2 x 30min with 0.5% triton X-100 in PBS. Sections were floated in PBS and mounted on slides with Vectashield (Vector Laboratories).

Ox3ZG construct

To generate the Ox3ZG construct, a genomic fragment from a *129 Sv/Ev* library containing the 3' end of the mouse *Cldn11* gene (Phage #2, Fig.2A [3]) was restriction endonuclease-digested with *Not I,* sub-cloned into a pUC2.2 plasmid [4] and further restriction-digested with *Fsp I* and *Not I* to remove an 11 kilobase fragment comprising the 3' end of intron 2, exon 3 and 5 kilobases of downstream sequence. This fragment was blunt-end sub-cloned into the *Nae I* site of a transgene expression cassette containing a 158 base pair thymidine kinase minimal promoter immediately upstream of the *lacZ* coding region and a genomic fragment from the human *beta-globin* gene containing exon 2 through exon 3 [5]. The resulting *pUC2.2-Ox3ZG* plasmid was digested with *Not I* and a 16 kilobase fragment purified from agarose for male pronuclear injection in single cell 0.5 day old mouse embryos. Mice were genotyped using a standard PCR protocol with the following primer pair in mouse *Cldn11* (5'- CCT TTG CAT CTG TCC TGG CCT CTG T -3') and *E.coli lacZ* (5'- ATG TGC TGC AAG GCG ATT AAG TTG G -3').

Transient transgenic mice

Pronuclear injections were performed according to institutional protocols at the University of Michigan Transgenic Animal Model core facility. Founder mice from independent Ox3ZG transgenic lines were perfused for 15min using 2% paraformaldehyde, 2mM $MgCl_2$ in 0.1M PIPES buffer, pH6.9, according to previously established protocols [5]. Brains were dissected, cut with a razor blade along the sagittal midline and permeabilized overnight with 0.01% deoxycholate (Sigma), 0.02% NP-40 (Sigma), and 2mM $MgCl_2$ in PBS, then incubated overnight at 37°C in permeabilization solution containing 17.5mM $K_3Fe(CN)_6$, 17.5mM $K_4Fe(CN)_6$, and 1mg/ml X-gal (5-bromo-4-chloro-3-hydroxyindole; Thermo Fisher Scientific). Whole mount stained brains were photographed using a Nikon SMZ1500 dissecting microscope equipped with an HR Plan Apo, WD 54 1X lens, and Sony DKC-5000 digital camera.

Auditory neurophysiology

Auditory brainstem responses (ABRs) and distortion product otoacoustic emissions (DPOAEs) were recorded from 2 month old (2M) avertin-anesthetized (375mg/kg) mice using Intelligent Hearing Systems equipment (ihsys, Miami, FL) according to previously established procedures [6].

Northern blotting

Whole brains from adult mice were rapidly dissected from decapitated mice and frozen in dry ice for storage at -80°C. Brains were partially thawed in 4M guanidinium chloride, 0.1M mercaptoethanol and homogenized (Tekmar tissuemizer, Tekman Co, Cincinnati, OH) for $CsCl_2$ purification as previously described [5,7]. Full-length *Cldn11* cDNA was used to probe the northern blots.

Tail suspension test

Mice were suspended in air by taping their tail to a horizontal rod which was elevated 12 inches above a table surface. Before applying the tape, a 2.5cm length of polypropylene tubing (loosely fitting) was placed over the tail to prevent mice from grabbing and crawling up their tail during testing. Mice were suspended for 6min and video recorded for subsequent analysis, where the total times spent immobile versus moving were determined.

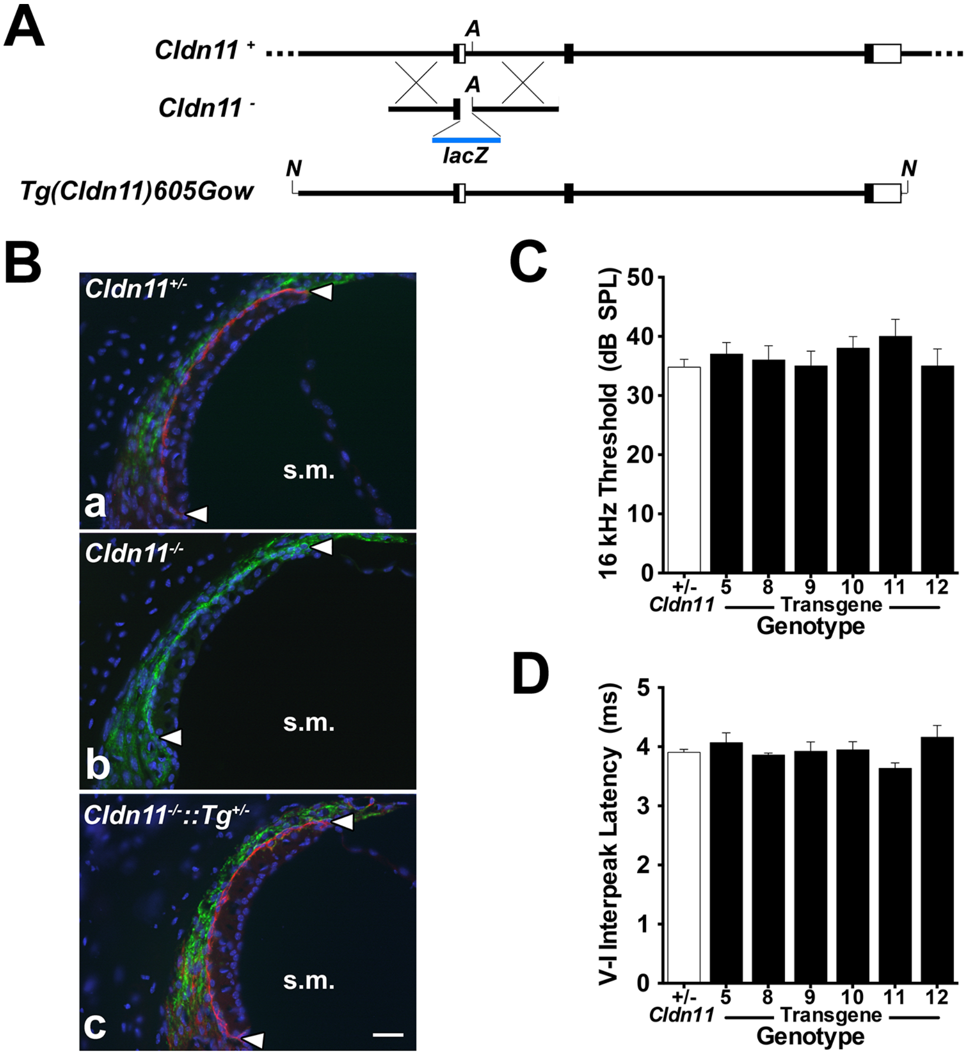

Supplementary Fig. S1 – Overexpressing Tg(Cldn11)605Gow in the cochlea is not toxic

(A) Schematic of the endogenous *Cldn11* gene (*Cldn11* $^{+}$) in mice showing three coding exons and a construct used to generate the *Cldn11*-null (*Cldn* $^{-}$) allele [3] as well as the region of the endogenous gene included in the Tg(Cldn11)605Gow transgene [5]. This transgene expresses in testis and basal cells of the cochlear lateral wall, consistent with endogenous gene expression. *A*, *Acc 65 I* restriction endonuclease site used to identify the transgene allele. *N*, *Not I* restriction sites used to excise the transgene from the plasmid for injection into male pronuclei of fertilized mouse embryos. (**B**) Confocal micrographs of the lateral wall of 2M control **(Ba)**, *Cldn11* $^{-/-}$ **(Bb)**, and *Cldn11* $^{-/-}$ *::Tg* $^{+/-}$ mice. Cryostat sections are labeled with DAPI (nuclei) and antibodies against connexin 26 (green) and claudin 11 (red). The claudin 11 labeling in **(Bc)** is specific for basal cells and closely resembles the endogenous claudin 11 staining in controls **(Ba)**. s.m., scala media. Arrowheads demarcate each end of the stria vascularis. (**C**) ABR thresholds measured in 2M control (+/-) and six independent lines of Tg(Cldn11)605Gow transgenic mice (lines #5, 8 – 12) harboring two functional alleles of the endogenous *Cldn11* gene. All transgenic lines have normal hearing thresholds for 16 kHz pure tone stimuli at 2 [$F_{(6,25)}$ = .634, p = .70] and 4M (not shown), indicating that overexpression of claudin 11 by at least two-fold [5] does not confer a significant phenotype. (**D**) The interpeak latencies of ABR waves V-I (as well as V-II, V-II and II-I) at 16 kHz are also comparable to controls ($F_{(6,27)}$ = 1.39, p = .26).

Absence of Claudin 11 in CNS Myelin Perturbs Behavior and Neurotransmitter Levels in Mice

Maheras KJ, Peppi M, Ghoddoussi F, Galloway MP, Perrine SA, Gow A

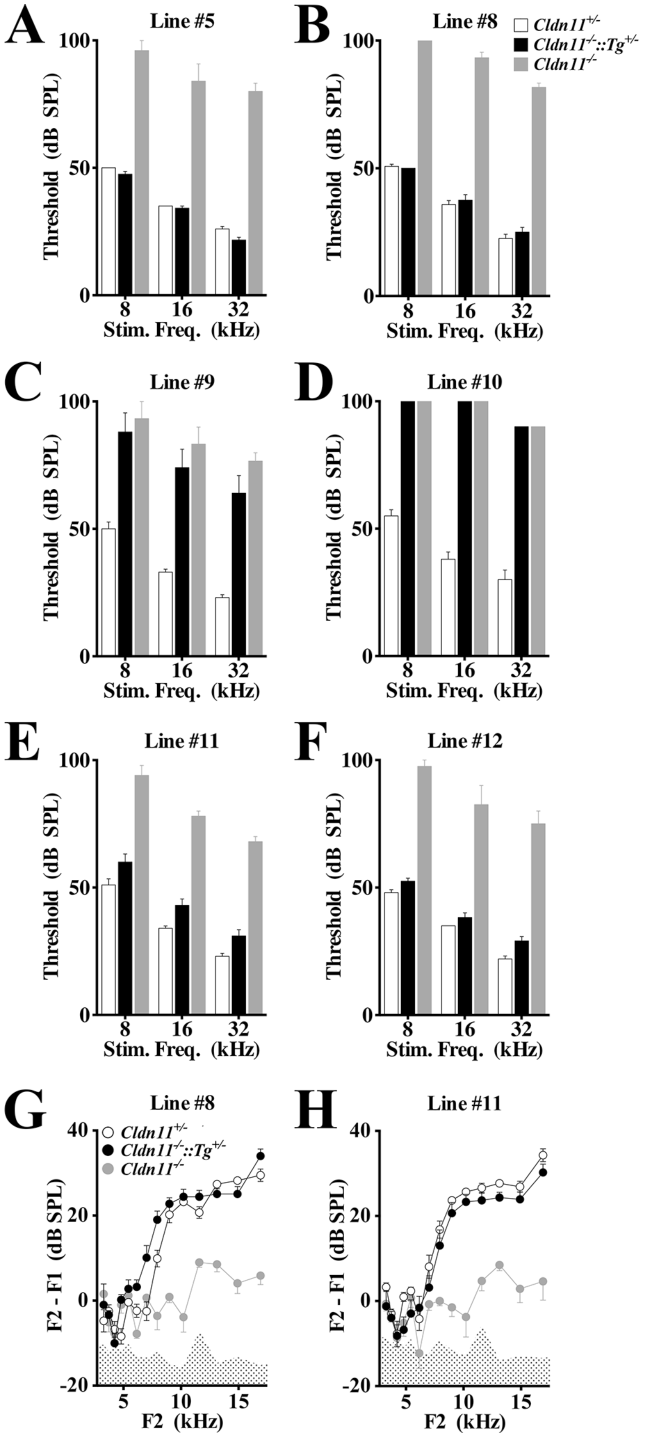

Supplementary Fig. S2 – Rescue of the peripheral deafness phenotype in *Cldn11*$^{-/-}$ mice by the Tg(Cldn11)605Gow transgene

(A-F) ABR thresholds for 8, 16, and 32 kHz pure tone stimuli from 2M transgenic mice on a *Cldn11*$^{-/-}$ genetic background (*Cldn11*$^{-/-}$*::Tg*$^{+/-}$). Transgene lines #5 **(A)**, 8, **(B)** 11 **(E)** and 12 **(F)** rescue the hearing thresholds to normal levels, while lines #9 **(C)** and 10 **(D)** are indistinguishable from *Cldn11*$^{-/-}$ mice. Lines #9 and 10 also fail to rescue the azoospermia phenotype in *Cldn11*$^{-/-}$ mice [5,8], probably because claudin 11 expression from the transgene is too low. (**G, H)** Distortion-product otoacoustic emissions (DPOAEs) are also rescued by transgene expression in basal cells (lines #8 and 11 measured) between 3 – 18 kHz compared to *Cldn11*$^{-/-}$ mice, which indicates that outer hair cell physiology is restored by the transgene. Hashed regions indicate noise floor.

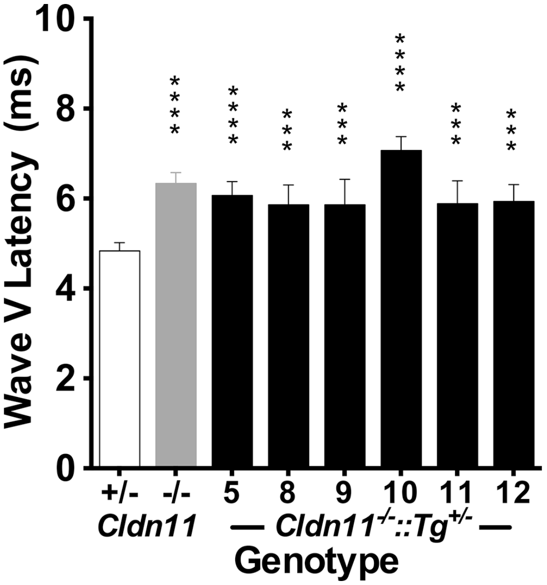

Supplementary Fig. S3 – ABR wave V latency is not rescued by the Tg(Cldn11)605Gow transgene

The latency of wave V of the ABR is commonly used to estimate the relative conduction velocity along myelinated fibers in the auditory pathway of a number of mammalian species including humans, but the transmission time of auditory signals in the brainstem is measured directly in the current study. The Tg(Cldn11)605Gow transgene rescues hearing threshold deficits and low endocochlear potential in *Cldn11*-null mice (Figure S2) associated with the loss of claudin 11 expression in the basal cells of the stria vascularis [6]. However, the transgene is not expressed in oligodendrocytes of $Cldn11^{-/-}::Tg^{+/-}$ mice and the transmission time defect persists in these animals [3,9,10]. One-way ANOVA $F_{(7,33)} = 12.04$; $p < .0001$; ***, $p < .001$; ****, $p < .0001$ for Bonferroni’s multiple comparisons tests.

Absence of Claudin 11 in CNS Myelin Perturbs Behavior and Neurotransmitter Levels in Mice

Maheras KJ, Peppi M, Ghoddoussi F, Galloway MP, Perrine SA, Gow A

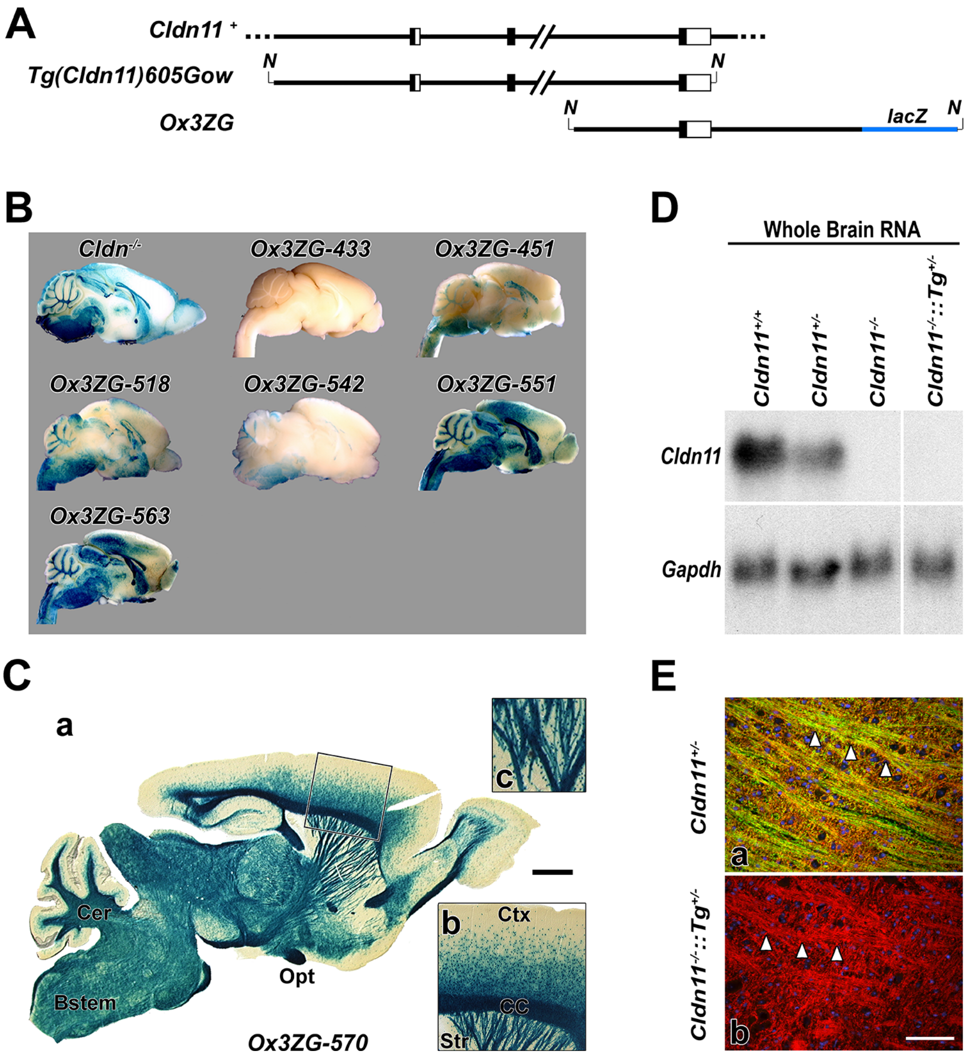

Supplementary Fig. S4 – The oligodendrocyte-specific *Cldn11* enhancer region is downstream of the Tg(Cldn11)605Gow transgene **(A)** Schematic of the endogenous *Cldn11* gene (*Cldn11* $^{+}$) in mice showing three coding exons and the region of the endogenous gene included in the Tg(Cldn11)605Gow transgene [5]. The exon 3 region (*Ox3ZG*, black) of the *Cldn11* gene was tethered to a minimal thymidine kinase promoter and *lacZ* reporter (blue) to test for the presence of oligodendrocyte-specific expression in transient transgenic mice [5]. **(B)** Transient transgenic mouse analysis of the *Ox3ZG* construct shows β-galactosidase expression in whole-mount brain preparations from 5 lines with an oligodendrocyte-specific pattern comparable to the positive control (top left), although expression in lines#451 and 542 is weak. Line#433 does not express the transgene, which may have been incorporated into heterochromatin in the injected embryo (approximately 20% of transgene insertions do not express). **(C)** Expression of β-galactosidase at low magnification **(a)** in a 100μm vibratome section from a 6th transient transgenic line. Expression is robust in all white matter tracts including brainstem (Bstem), cerebellum (Cer) and optic nerve (Opt). At higher magnification **(b)**, β-galactosidase expression is visible in cortex (Cor), corpus callosum (CC) and striatum (Str). Oligodendrocyte-specific expression is particularly obvious in striatum **(c)**, where these cells reside externally and extend processes into the axon bundles they ensheath. **(D)** Whole brain total RNA samples from wild type (*Cldn11* $^{+/+}$), *Cldn11* $^{+/-}$, *Cldn11* $^{-/-}$ and *Cldn11* $^{-/-}$*::Tg* $^{+/-}$ mice were northern blotted and probed with full-length mouse *Cldn11* cDNA. These samples were run on the same gel, but the *Cldn11* $^{-/-}$ and *Cldn11* $^{-/-}$*::Tg* $^{+/-}$ samples were separated by several lanes. The blot demonstrates that *Cldn11* shows gene copy-number dependent expression in mice with 2, 1 or 0 copies. The Tg(Cldn11)605Gow transgene shows no expression in CNS. **(E)** Immunofluorescence labeling of sagittal cryostat sections of auditory brainstem from 8 month *Cldn* $^{+/-}$ **(a)** and *Cldn11* $^{-/-}$*::Tg* $^{+/-}$ **(b)** mice with antibodies against Claudin 11 (green) and MBP (red) show that transgene-derived claudin 11 is not expressed in myelinated fibers (arrowheads) of the trapezoid body. Nuclei are labeled with DAPI (blue). Scale bar: 100μm.

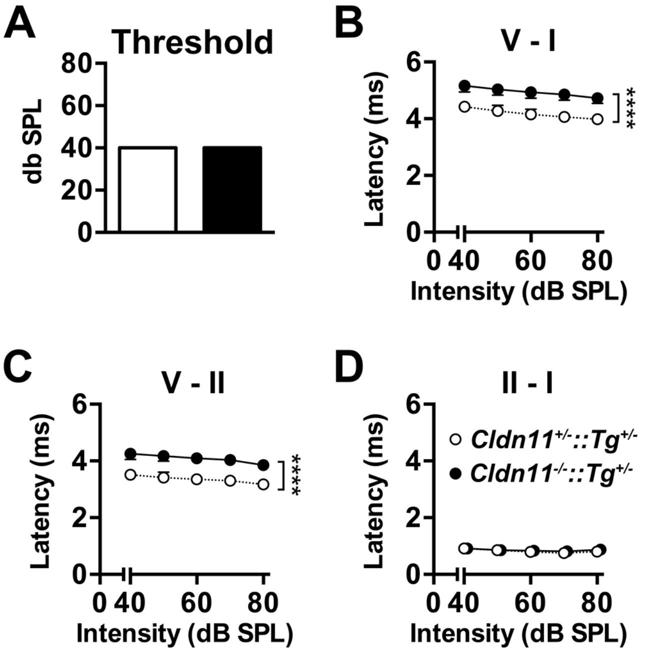

Supplementary Fig. S5 – Increased interpeak latencies for central waves of *Cldn11*$^{-/-}$::*Tg*$^{+/-}$ mice characterized in the current study

(A) ABR thresholds for *Cldn11*$^{-/-}$::*Tg*$^{+/-}$ and *Cldn11*$^{+/-}$::*Tg*$^{+/-}$ mice at similar at 32 kHz. The thresholds for all mice tested was 40 dB SPL. **(B)** Calculated interpeak latencies for *Cldn11*$^{-/-}$::*Tg*$^{+/-}$ and *Cldn11*$^{+/-}$::*Tg*$^{+/-}$ mice. **(Ba)** Total interpeak latency, wave V minus I is significantly greater in *Cldn11*$^{-/-}$::*Tg*$^{+/-}$ mice [$F_{(1,20)}$ = 85.11, p < .0001]. **(Bb)** Central interpeak latency components, wave V minus II remains significantly increased in *Cldn11*$^{-/-}$::*Tg*$^{+/-}$ mice [$F_{(1,20)}$ = 125.7, p < .0001]. **(Bc)** Peripheral interpeak latency, wave II minus I is similar between *Cldn11*$^{+/-}$::*Tg*$^{+/-}$ and *Cldn11*$^{-/-}$::*Tg*$^{+/-}$ mice [$F_{(1,20)}$ = .60, p = .45]. Data points for *Cldn11*$^{-/-}$::*Tg*$^{+/-}$ mice in **Bc** are right-shifted on the abscissa for clarity. Data plotted as mean ± SEM; 10 ≤ n ≤ 12; ****, p < .0001.

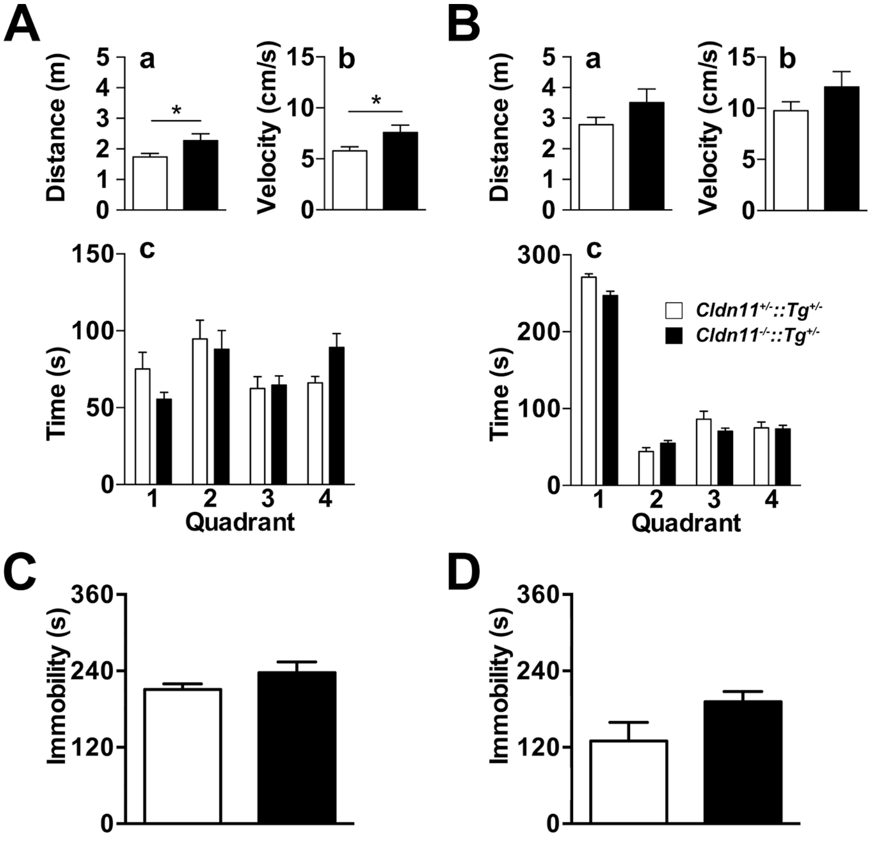

Supplementary Fig. S6 – Additional parameters from OF and tail suspension tests Open field testing of $Cldn11^{+/-}::Tg^{+/-}$ and $Cldn11^{-/-}::Tg^{+/-}$ mice at 2M **(A)** and 7M **(B)**. **(Aa)** Total distance traveled by $Cldn11^{-/-}::Tg^{+/-}$ mice exceeds that for $Cldn11^{+/-}::Tg^{+/-}$ mice and indicates these mice were active in the OF arena during testing [$t_{(18)} = 2.15$, $p = .045$]. **(Ab)** Average velocity for $Cldn11^{-/-}::Tg^{+/-}$ mice exceeds that for $Cldn11^{+/-}::Tg^{+/-}$ mice which is consistent with the distance data [$t_{(18)} = 2.15$, $p = .045$]. **(Ac)** The quadrant preference in the arena is comparable between the genotypes indicating that the local environment did not play a significant role in performance of the mice [$F_{(1,18)} = 3.50$, $p = .08$]. **(Ba)** $Cldn11^{+/-}::Tg^{+/-}$ and $Cldn11^{-/-}::Tg^{+/-}$ mice travel similar distances during OF testing [$t_{(17)} = 1.50$, $p = .15$]. **(Bb)** Average velocities of $Cldn11^{+/-}::Tg^{+/-}$ and $Cldn11^{-/-}::Tg^{+/-}$ mice are similar [$t_{(17)} = 1.28$, $p = .22$]. **(Bc)** A strong bias for quadrant 1 is apparent in these experiments, perhaps associated with uneven lighting in the arena, but there are no differences between $Cldn11^{+/-}::Tg^{+/-}$ and $Cldn11^{-/-}::Tg^{+/-}$ mice [$F_{(1,17)} = 4.24$, $p = .06$]. **(C,D)** Tail suspension tests of six minutes duration demonstrate that $Cldn11^{-/-}::Tg^{+/-}$ mice perform comparably to controls and do not exhibit a depression-like endophenotype at 2M **(C)** or 7M **(D)** [2M: $t_{(18)} = 1.38$, $p = .19$; 7M: $t_{(17)} = 1.87$, $p = .09$]. Data plotted as mean ± SEM, $9 \le n \le 10$; * = $p < .05$.

References

1 Doucet, J. R. & Ryugo, D. K. Projections from the ventral cochlear nucleus to the dorsal cochlear nucleus in rats. *J Comp Neurol* **385**, 245-264 (1997).

2 Doucet, J. R. & Ryugo, D. K. Axonal pathways to the lateral superior olive labeled with biotinylated dextran amine injections in the dorsal cochlear nucleus of rats. *J Comp Neurol* **461**, 452-465 (2003).

3 Gow, A. *et al.* CNS myelin and sertoli cell tight junction strands are absent in Osp/claudin-11 null mice. *Cell* **99**, 649-659 (1999).

4 Gow, A., Friedrich, V. L., Jr. & Lazzarini, R. A. Myelin basic protein gene contains separate enhancers for oligodendrocyte and Schwann cell expression. *J Cell Biol* **119**, 605-616 (1992).

5 Wu, X. *et al.* Transgene-mediated rescue of spermatogenesis in Cldn11-null mice. *Biol Reprod* **86**, 131-139 (2012).

6 Gow, A. *et al.* Deafness in Claudin 11-null mice reveals the critical contribution of basal cell tight junctions to stria vascularis function. *J Neurosci* **24**, 7051-7062 (2004).

7 Chirgwin, J. M., Przybyla, A. E., MacDonald, R. J. & Rutter, W. J. Isolation of biologically active ribonucleic acid from sources enriched in ribonuclease. *Biochemistry* **18**, 5294-5299 (1979).

8 Mazaud-Guittot, S. *et al.* Claudin 11 deficiency in mice results in loss of the Sertoli cell epithelial phenotype in the testis. *Biol Reprod* **82**, 202-213. PMC2802123 (2010).

9 Denninger, A. R. *et al.* Claudin-11 Tight Junctions in Myelin Are a Barrier to Diffusion and Lack Strong Adhesive Properties. *Biophys J* **109**, 1387-1397 (2015).

10 Gow, A. & Devaux, J. A model of tight junction function in central nervous system myelinated axons. *Neuron Glia Biol* **4**, 307-317 (2008).
